# Genome-wide analysis of spatiotemporal expression patterns during rice leaf development

**DOI:** 10.1186/s12864-021-07494-5

**Published:** 2021-03-09

**Authors:** Masayuki Miya, Takanori Yoshikawa, Yutaka Sato, Jun-Ichi Itoh

**Affiliations:** 1 Graduate School of Agricultural and Life Sciences, University of Tokyo, Tokyo, 113-8657 Japan; 2 Graduate School of Agriculture, Kyoto University, Kyoto, 606-8502 Japan; 3 Institute of Crop Science, National Agriculture and Food Research Organization (NARO), Tsukuba, 305-8518 Japan

**Keywords:** Rice, Leaf development, Leaf blade, Leaf sheath, Blade-sheath boundary, Transcriptome, In situ hybridization

## Abstract

**Background:**

Rice leaves consist of three distinct regions along a proximal-distal axis, namely the leaf blade, sheath, and blade-sheath boundary region. Each region has a unique morphology and function, but the genetic programs underlying the development of each region are poorly understood. To fully elucidate rice leaf development and discover genes with unique functions in rice and grasses, it is crucial to explore genome-wide transcriptional profiles during the development of the three regions.

**Results:**

In this study, we performed microarray analysis to profile the spatial and temporal patterns of gene expression in the rice leaf using dissected parts of leaves sampled in broad developmental stages. The dynamics in each region revealed that the transcriptomes changed dramatically throughout the progress of tissue differentiation, and those of the leaf blade and sheath differed greatly at the mature stage. Cluster analysis of expression patterns among leaf parts revealed groups of genes that may be involved in specific biological processes related to rice leaf development. Moreover, we found novel genes potentially involved in rice leaf development using a combination of transcriptome data and in situ hybridization, and analyzed their spatial expression patterns at high resolution. We successfully identified multiple genes that exhibit localized expression in tissues characteristic of rice or grass leaves.

**Conclusions:**

Although the genetic mechanisms of leaf development have been elucidated in several eudicots, direct application of that information to rice and grasses is not appropriate due to the morphological and developmental differences between them. Our analysis provides not only insights into the development of rice leaves but also expression profiles that serve as a valuable resource for gene discovery. The genes and gene clusters identified in this study may facilitate future research on the unique developmental mechanisms of rice leaves.

**Supplementary Information:**

The online version contains supplementary material available at 10.1186/s12864-021-07494-5.

## Background

Leaves, which are the main site of photosynthesis in higher plants, are usually polarized along three axes: proximal-distal, adaxial-abaxial, and medial-lateral. Tissues arranged along these axes have characteristic morphologies and functions. As leaves are derived from immature cell populations protruding from the shoot apical meristems (SAM), their morphology and functions must be acquired during the course of development. Leaf development is a tightly orchestrated process incorporating multiple events crucial to organogenesis: axis determination, pattern formation, and identity establishment. Additionally, the growth of leaf primordia, which relies on cell proliferation and differentiation, is precisely regulated both temporally and spatially to produce typically shaped leaves.

The morphology of leaves varies greatly among species and developmental phases and environments, and this variation is driven by differences in leaf genetic programs among species. Hence, the mechanisms regulating leaf morphogenesis should be studied in a wide variety of species. Most information currently available has been obtained from analyses of the model eudicot plant *Arabidopsis*. A number of genes regulating leaf development have been identified in *Arabidopsis* [1], and the molecular mechanisms of leaf morphogenesis in various species have been elucidated based on information obtained from *Arabidopsis*.

**Fig. 1:**
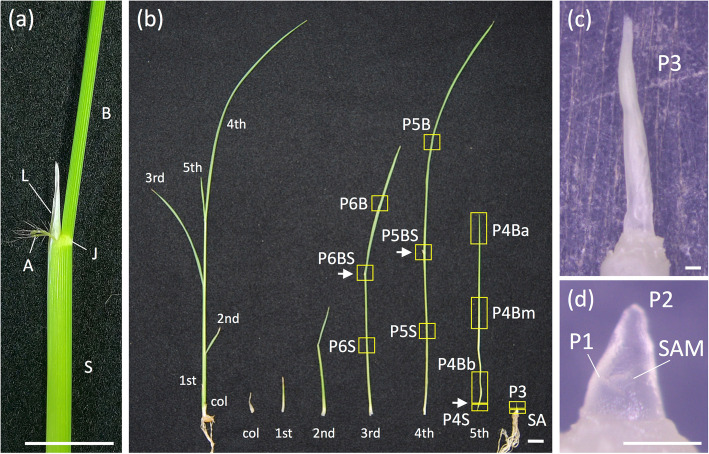
Overview of the samples used for microarray analysis. **a** Morphology of mature rice leaves. A, auricle; B, leaf blade; J, lamina joint; L, ligule; S, leaf sheath. **b** Samples used for microarray analysis. A seedling and the separated leaves of the seedling are shown. Yellow boxes indicate the 12 parts sampled for microarray analysis. Arrows show the boundaries between the leaf blade and sheath. See Table 1 for details. **c** Magnified view of the leaf primordium at stage P3. **d** Magnified view of the shoot apex containing the shoot apical meristem and P1 and P2 leaf primordia. Scale bars: 1 cm in (**a**), (**b**); 20 μm in (**c**), (**d**)

Grasses belong to the monocot clade, and their leaf morphology is distinct from that of *Arabidopsis*. Although grass leaves are polarized along the same three axes as those of other plants, they are unique in that distinct regions with differing morphology and function are located along the proximal-distal axis (Fig. 1a). The leaf blade is the distal part of the leaf; it has a flat structure and is rich in mesophyll cells, in which photosynthesis occurs. The leaf sheath is located at the basal part of the leaf and has a thick structure that protects inner leaves and provides structural support to the blade. The boundary region between the blade and sheath comprises the lamina joint, ligule, and auricle. The lamina joint acts as a hinge that allows the blade to bend abaxially, thereby optimizing light capture by the blade. Each of the three regions undergoes different developmental processes. In addition, spatiotemporal coordination of tissue differentiation during development contributes to the final leaf morphology. As with *Arabidopsis*, tissue differentiation in grass leaves proceeds in the basipetal direction, suggesting that these processes are under precise spatial and temporal control by genetic mechanisms.

To date, several genes that are important for grass leaf morphology have been identified. Related to the organs and tissues that are differentiated along the proximal-distal axis, *LIGULELESS1*, a member of the *SQUAMOSA PROMOTER BINDING-LIKE* (*SPL*) gene family, is essential for the differentiation of organs in the blade-sheath boundary region [2–4]. Homologs of *Arabidopsis BLADE-ON-PETIOLE* genes in rice are important for sheath development [5]. Class I *KNOX* genes important for the maintenance of the SAM are believed to provide proximal cues for leaf primordia [6, 7]. Meanwhile, cell proliferation patterns have been reported to affect grass leaf morphology. Cell proliferation in immature tissues of leaf primordia is controlled by protein complexes encoded by two gene families, *GROWTH-REGULATING FACTORS* (*GRF*s) and *GRF-INTERACTING FACTORS* (*GIF*s) [8–11]. Changes in their protein complex composition reportedly serve as a switch for the transition from cell division to cell expansion [12]. However, few of the genes that play important roles in grass leaf development have been identified.

For elaboration of leaf development and morphology, gene expression should be precisely regulated both temporally and spatially. Thus, revealing the expression patterns of genes during development would contribute significantly to understanding the genetic mechanisms behind the process of leaf establishment. Transcriptome analysis is a powerful method for exploring gene expression dynamics at both the genome-wide and single-gene levels. To date, transcriptome analysis of leaf development has been performed in species including *Arabidopsis* [13, 14], maize [15–22], and rice [23–25]. In particular, transcriptome changes accompanied by tissue differentiation have been intensively studied in the developing leaf blade in maize. However, no study to date has reported the temporal transcriptomic changes occurring from leaf initiation to leaf maturation in rice. Furthermore, most transcriptome studies of grass leaves have been performed on tissues from only one of the regions along the longitudinal axis, making it difficult to draw direct comparisons among regions. Therefore, to fully elucidate gene expression profiles and characterize gene function, it is necessary to investigate spatiotemporal changes in leaf transcriptomes from each region along the longitudinal axis of the leaf.

In this study, we performed transcriptome analysis of rice leaf development using the Agilent rice 44 K microarray, which is compatible with the rice expression database RiceXPro [26, 27]. Our experimental design included a broad range of developmental stages and several distinct regions along the leaf longitudinal axis, which allowed us to capture overall transcriptome dynamics throughout leaf development. Our data analysis uncovered trends in the expression patterns of certain gene clusters during leaf development and revealed relationships between developmental events and those gene clusters. In addition, we performed in situ hybridization with 49 selected genes based on the data from our transcriptome analysis. As a result, we identified multiple genes with localized expression in tissues characteristic of grass leaves. The present work provides a foundation for future analyses of genes with novel functions in rice leaf development.

## Results

### Experimental design for microarray dataset

Rice leaf ontogeny, i.e., the developmental process from initiation to maturation, is described in Itoh et al. (2005) [28]. Briefly, according to the staging system based on plastochron numbers (Pn), the P1 leaf primordium protrudes from the SAM and then grows to surround the SAM at stage P2. During the P1 and P2 stages, the leaf primordium consists of undifferentiated cells with no morphological characters. During the P3 stage, the boundary between the blade and sheath is established, and the future blade and sheath parts can be distinguished. In addition, the ligule primordium is formed in the boundary region at this stage. Although most of the P3 leaf primordium is comprised of undifferentiated cells, the outermost cells on the distal side of the primordium begin to differentiate into epidermal cells. During stage P4, the leaf blade elongates rapidly, and the difference between the blade and sheath becomes more pronounced. The P4 leaf primordium exhibits a clear gradient of cell states along its longitudinal axis; cells in the proximal region remain undifferentiated, whereas those in the distal region are differentiated. During stage P5, the leaf sheath elongates rapidly, and the growth and maturation of the leaf are completed by the P6 stage, whereas bending of the lamina joint occurs between stages P5 and P6.

To obtain a comprehensive transcriptome of leaf development in rice, we sampled 12 leaf parts representing various stages and components along the longitudinal axis (Fig. 1b–d; Table 1). Rice seedlings at the four-leaf stage were dissected into 12 parts: shoot apex containing the SAM and P1 and P2 leaf primordia (Fig. 1d); entire P3 leaf primordium (Fig. 1c); apical, middle, and proximal parts of P4 leaf blade; P4 leaf sheath; and the leaf blade, sheath, and boundary region of P5 and P6 leaves. Three biological replicates were prepared for each part, and their RNA was hybridized to a 44 K rice microarray (Agilent Technologies, Santa Clara, CA) [26, 29, 30]. Out of 43,144 probes corresponding to 29,864 genes on the rice 44 K microarray platform, 31,996 probes corresponding to 24,022 genes were expressed in at least one sample. Normalized expression levels of these 31,996 probes corresponding to 24,022 genes were used in this study. Distribution of the normalized expression levels of those probes for each sample roughly exhibited the normal distribution centered at zero (Supplemental Figure 1). Pearson correlation analysis showed strong correlations among the three replicates, indicating that our dataset was highly reproducible (Supplemental Figure 2). Furthermore, we verified expression profiles of six genes in P4 stage by real-time RT-PCR analysis (Supplemental Figure 3). The expression patterns of all the genes were consistent with the patterns from microarray analysis. This suggests that our expression data of microarray is considerably accurate and reliable.

**Table 1:** Description of samples used for microarray analysis

Sample	Stage	Tissue	Abbreviation
Shoot Apex	P0, P1, P2	Shoot apex containing SAM and P1 and P2 leaf primordia	SA
P3	P3	Whole P3 leaf primordia	P3
P4Sheath	P4	Leaf sheath	P4S
P4Blade_basal	P4	Basal part of leaf blade	P4Bb
P4Blade_middle	P4	Middle part of leaf blade	P4Bm
P4Blade_apical	P4	Apical part of leaf blade	P4Ba
P5Sheath	P5	Leaf sheath	P5S
P5Boundary	P5	Boundary region between leaf blade and sheath	P5BS
P5Blade	P5	Leaf blade	P5B
P6Sheath	P6	Leaf sheath	P6S
P6Boundary	P6	Boundary region between leaf blade and sheath	P6BS
P6Blade	P6	Leaf blade	P6B

### Transcriptome dynamics during rice leaf development

**Fig. 2:**
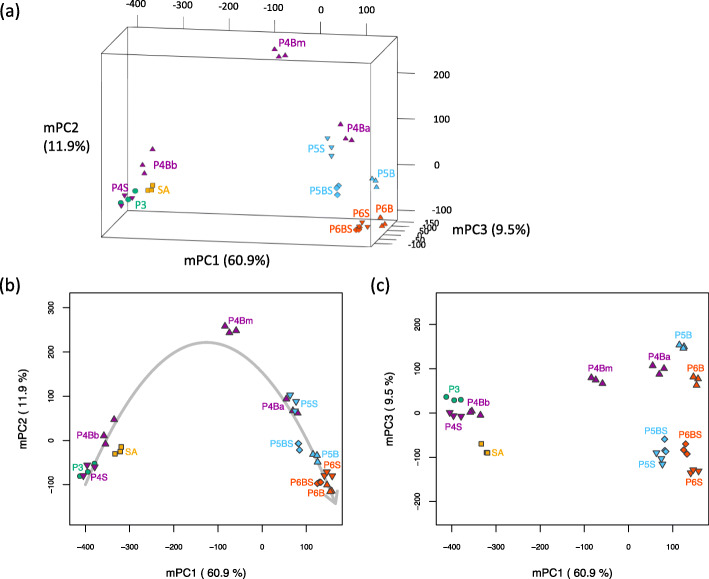
Principal component analysis (PCA) score plot of samples based on modified principal components. **a** The space defined by mPC1, mPC2, and mPC3. A three-dimensional model allowing interactive rotation is available in additional file 4. See Supplemental Figure 4 for the PCA score plot based on the original principal components. **b** The space defined by mPC1 and mPC2. The gray arrow represents the regression curve for the distribution of samples in this space. **c** The space defined by mPC1 and mPC3. The proportions of the total variance explained by mPC1, mPC2, and mPC3 are shown in parentheses. Samples collected at the same stage are shown in the same color. Samples with different tissue identities are indicated by different symbols: shoot apex, square; P3 leaf, circle; blade, triangle; blade-sheath boundary, diamond; sheath, inverted triangle

To elucidate the transcriptome dynamics that occur during leaf development, principal component analysis (PCA) was performed using all samples. The first, second, and third principal components (PC1, PC2, and PC3) explained 60.9, 13.2, and 8.1% of the total variance among samples, respectively (Supplemental Figure 4; Additional file 3). Plotting the samples within the three-dimensional space defined by PC1, PC2, and PC3 allowed the relationships among the samples to be visualized, reflecting the two properties of tissue differentiation state and tissue identity (Supplemental Figure 4a; Additional file 3). However, these properties were poorly represented by each principal component (Supplemental Figure 4b and c) due to the fact that PC2 was excessively attracted toward P4Bm, which was located distant from the other samples. Generally, PCA is not robust to outliers, and principal components tend to be attracted toward them, interfering with the detection of the overall dataset structure. To avoid excessive attraction of PC2 toward the outlier P4Bm, we modified PC1, PC2, and PC3 while retaining positional relationships between the samples, as follows. PC1, PC2, and PC3 scores were treated as variables, and PCA was again applied using all samples except P4Bm, resulting in modified principal components (mPC1, mPC2, and mPC3) that were not excessively affected by P4Bm. Altogether, mPC1, mPC2, and mPC3 explained 60.9, 11.9, and 9.5% of the total variance among samples, respectively, and successfully captured the characteristic patterns of the dataset (Fig. 2; Additional file 4).

Two groups were separated by mPC1, namely, immature tissues represented by SA, P3, P4S, and P4Bb, mature tissues represented by P4Ba, and samples derived from P5 and P6 stage leaves. This result suggests that mPC1 represented the differences between immature and mature tissues (Fig. 2b). Conversely, mPC2 characterized samples with intermediate tissue differentiation, most notably P4Bm, suggesting that mPC2 represented the transient state of the transcriptome during tissue differentiation (Fig. 2b). Thus, an arrow fitting the distribution of the samples in the space defined by mPC1 and mPC2 would represent the change in transcriptome dynamics associated with tissue differentiation from the immature state through the transient state to the mature state. Collectively, mPC1 and mPC2 explained 72.8% of the total variance among samples, suggesting that tissue differentiation state has profound effects on the leaf transcriptome. Moreover, samples derived from the P4 leaf exhibited large transcriptomic variations, whereas all P4-stage leaf samples including sheath samples were aligned along the arrow. This result indicates that the shift in the transcriptome associated with leaf maturation is found throughout the leaf during P4, coinciding with intensive basipetal tissue differentiation at stage P4 [31].

In addition, mPC3 separated samples of the leaf blade—P4Bm, P4Ba, P5B, and P6B—from those of the leaf sheath and blade-sheath boundary region—P5S, P6S, P5BS, and P6BS, indicating that mPC3 represented differences between the leaf blade and sheath (Fig. 2c). On the other hand, only slight differences were observed among immature leaf samples such as P3, P4S, and P4Bb, suggesting that the transcriptomic difference between the leaf blade and sheath becomes more pronounced during maturation.

Overall, our results suggest that the transcriptome of each part of the leaf changes with the progression of tissue differentiation and the acquisition of tissue identity.

### Gene expression patterns during leaf development and their associations with gene function and transcriptional regulation

To uncover the major gene expression patterns during rice leaf development, we conducted cluster analysis of genes based on their expression patterns. Prior to cluster analysis, analysis of variance (ANOVA) was applied to detect differentially expressed genes among different parts of the leaf. Of 31,996 probes corresponding to 24,022 genes, 31,043 probes corresponding to 23,350 genes were extracted (*p*-value = 0.001 when adjusted for the false discovery rate [FDR]). K-means clustering was performed on these probes, and 28 clusters with distinct expression patterns were obtained (Supplemental Figure 5; Supplemental Table 1). For some of the clusters, there were large differences in expression levels among samples and characteristic expression patterns, suggesting that some groups of genes undergo similar changes in gene expression, and that such changes are associated with events during leaf development.

**Fig. 3:**
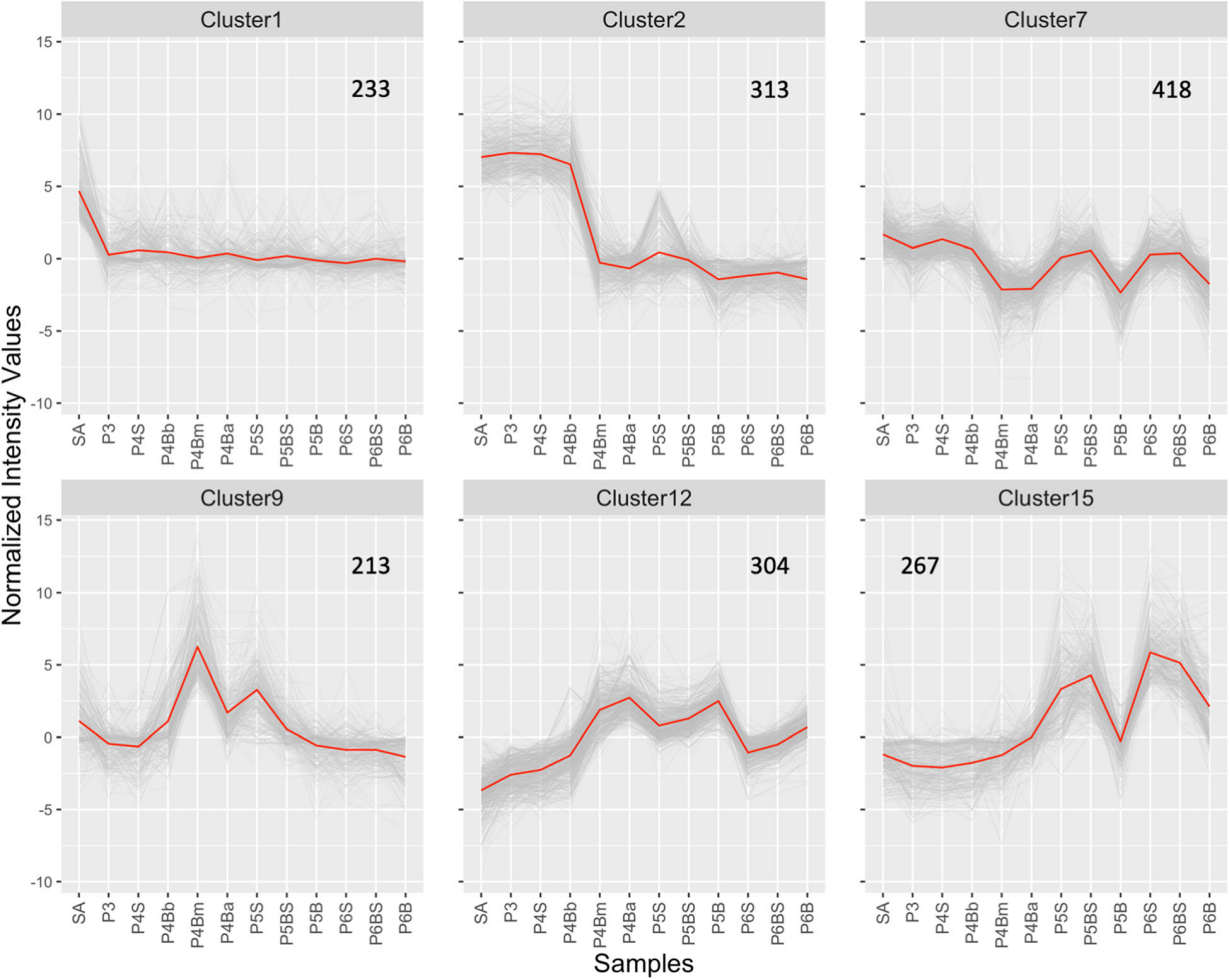
Six clusters showing expression patterns supposedly associated with events during leaf development. Grey lines indicate the expression profiles of probes in each cluster. Red lines indicate the mean of all probes in each cluster. Three biological replicates are summarized by median. The number of genes in each cluster is indicated in the upper right of each panel. See Supplemental Figure 5 for all 28 clusters obtained through K-means clustering

To evaluate how the gene expression patterns and dynamics of these gene clusters are related to the functions of the genes, we conducted Gene Ontology (GO) enrichment analysis on each cluster (Supplemental Figure 6). In addition, given the importance of transcriptional regulation to development, transcription factors and transcriptional regulators were extracted from each cluster (Supplemental Figure 7). These analyses identified the characteristic functions of genes within each cluster, including several genes that may be involved in specific processes during rice leaf development (Fig. 3; Table 2).

**Table 2:** Enriched GO terms, transcription factors, and transcriptional regulators in the six clusters in Fig. 3. See Supplemental Figures 6 and 7 for enriched GO terms and TF/TRs in all 28 clusters obtained through K-means clustering, respectively

Cluster	Enriched GO terms	TF/TRs
Cluster1	regulation of transcription, *p* = 4.8e-07	*OSH1/6/15/71* (ClassI *KNOX*), *OsNAM/OsCUC3*, *OsPLT2/3/4/5/6* (*BBM* clade *PLETHORA*), *OsTB1*
Cluster2	microtubule-based movement, *p* = 8.6e-05	*OsPLT1/7/8/9* (*ANT* clade *PLETHORA*), *OsGRF1/6/7/9/10*, *OsGIF1/MKB3*
Cluster7	main pathways of carbohydrate metabolism, *p* = 3.7e-05	*OsBOP1/2/3*
Cluster9	response to oxidative stress, *p* = 4.2e-07	
Cluster12	photosynthesis, *p* = 7.9e-21	*OsBBX8/10/12/17* (*C2C2-CO-like*), *OsPIL12/13* (*PIF*)
	carbon utilization by fixation of carbon dioxide, *p* = 8.5e-10	
	electron transport, *p* = 3.3e-07	
Cluster15	protein amino acid phosphorylation, *p* = 6.2e-05	

Cluster 1, a group of genes that was specifically expressed in the shoot apex, was enriched in genes involved in transcriptional regulation (regulation of transcription, *p* = 4.8e-07). Within this cluster were class I *KNOX* genes, which are important for SAM maintenance [32], and *OsNAM/OsCUC3* genes, which may be involved in organ boundary formation [33]. Thus, Cluster 1 was predicted to include genes related to SAM function and leaf initiation. This cluster also included genes in the *BBM* clade of the *PLETHORA* family [34], which are expressed in crown root primordia [35], and *OsTB1*, which is expressed in axillary buds [36]. Because the shoot apex tissue used in this study contained stem tissue as well as the SAM and leaf primordia, the presence of root- and axillary bud-related genes in this cluster was not surprising.

Cluster 2 contains genes that were highly expressed in tissues undergoing active cell proliferation. In this cluster, GO terms associated with cell division and cytokinesis (microtubule-based movement, *p* = 8.6e-05) were detected. Moreover, it contained *ANT* clade genes of the *PLETHORA* family [34], *GRF* family genes [12], and *OsGIF1/MKB3* [11], which have been described as promoters of cell proliferation in leaf primordia. Thus, Cluster 2 was expected to contain important genes related to cell proliferation in leaf primordia.

Cluster 9 genes were highly expressed in the middle parts of the P4 leaf blade and P5 leaf sheath. GO analysis revealed that this cluster contains class III peroxidases (response to oxidative stress, *p* = 4.2e-07). Some class III peroxidases regulate reactive oxygen species homeostasis in the apoplast, thereby affecting cell-wall stiffness [37, 38]. GO terms for cell-wall remodeling enzymes including XTHs (carbohydrate metabolism, *p* = 1.3e-3) [39] were also enriched in Cluster 9. Thus, Cluster 9 appears to be enriched in genes involved in the control of cell-wall extensibility and cell elongation.

Cluster 12 genes were mostly expressed at high levels in the mature leaf blade. GO terms for genes involved in photosynthesis (photosynthesis, *p* = 7.9e-21; carbon utilization by fixation of carbon dioxide, *p* = 8.5e-10; and electron transport, *p* = 3.3e-07) were enriched. This cluster included *C2C2-CO-like* family genes [40] and *phytochrome-interacting bHLH factors* (*PIF*s) [41]. Thus, Cluster 12 was expected to contain a high concentration of genes associated with photosynthesis and light-mediated signal transduction.

Cluster 7 genes were highly expressed in immature samples and samples from mature tissues from the sheath and blade-sheath boundary region. This cluster was enriched in genes involved in carbohydrate metabolism (main pathways of carbohydrate metabolism, *p* = 3.7e-05). The leaf sheath is believed to act as sink tissue for carbohydrates prior to heading [42]. In addition, this cluster includes *OsBOP* genes that are important for sheath development [5]. Thus, Cluster 7 was enriched in genes involved in the carbohydrate sink function and sheath development.

Cluster 15 consisted of genes that were preferentially expressed in the mature sheath and blade-sheath boundary region. This cluster was enriched in genes related to GO terms for protein kinases (protein amino acid phosphorylation, *p* = 6.2e-05). The blade-sheath boundary contains the lamina joint, which bends between stages P5 and P6. Various phytohormones and environmental stressors affect the bending process [43, 44], and many protein kinases exhibit temporal changes in expression during lamina-joint bending [25]. Thus, we assumed that Cluster 15 contains genes involved in lamina-joint bending.

Taken together, these analyses revealed genome-wide gene expression patterns during rice leaf development, and relationships between expression pattern and function were found in certain gene clusters.

### Identification of genes with localized expression during leaf development

To identify novel genes that play important roles in early development and subsequent morphogenesis and tissue formation in the rice leaf, we selected genes from Clusters 1 to 8 using K-means clustering analysis. These clusters contain genes that tended to be highly expressed during the early stages and weakly expressed during the later stages, and thus were expected to include candidate genes. Forty-nine genes, most of them involved in transcriptional regulation, were selected from the gene clusters, and their spatial expression patterns around the shoot apex were examined through in situ hybridization. Examples of these gene expression patterns are described below.

**Fig. 4:**
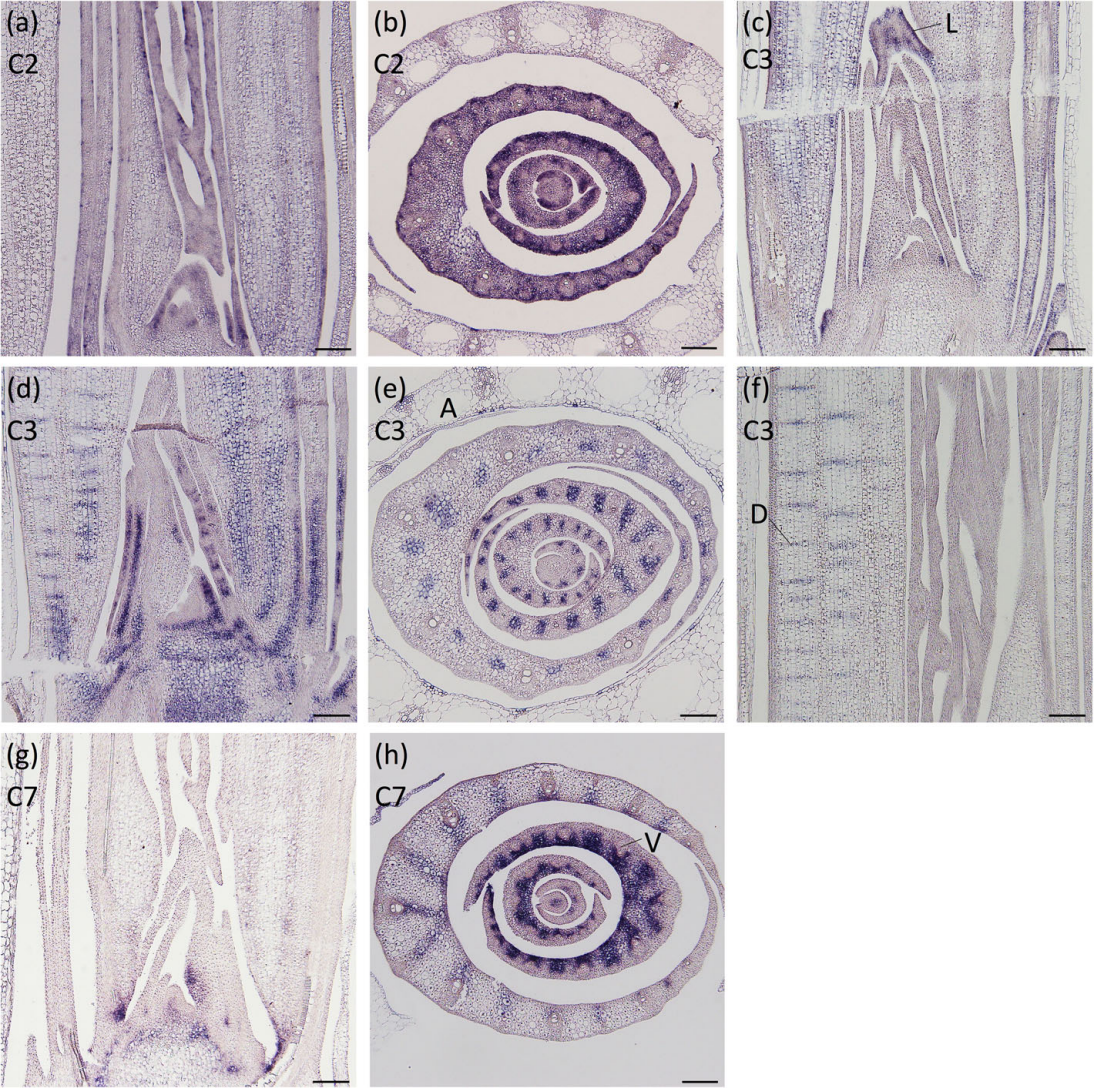
Spatial expression patterns of several genes identified through K-means clustering analysis. The expression patterns of (**a**, **b**) *Os02g0172800*, **c** *OsbHLH080*, **d**, e *OsbHLH166*, **f** *Os05g0363500*, and **g**, **h** *OsGH3–4* around the shoot apex. **a**, **c**, **d**, **f**, and **g** are longitudinal sections of the shoot apices, while (**b**), **e**, and **h** are transverse sections of the shoot apices. The cluster number to which each gene belongs is indicated in the upper left. L, ligule; A, aerenchyma; D, diaphragm; V, vascular. Scale bars: 100 μm

Cluster 2 included the *PLATZ* family transcription factor *Os02g0172800*, which is a co-ortholog of *ORESARA15* [45]. This gene was expressed in the basal part of immature leaves, and its expression was strongest in the abaxial side of the leaf primordium (Fig. 4a, b).

Tissue-specific expression patterns of three genes in Cluster 3 were detected. Expression of *OsbHLH080*, a member of the *bHLH* gene family, was observed in the abaxial base of leaf primordia and developing ligules (Fig. 4c). Another *bHLH* gene*, OsbHLH166,* was expressed mainly in the presumptive lysigenous aerenchyma areas of leaf primordia (Fig. 4d, e). *Os05g0363500*, which encodes a WD40 repeat-containing protein, was expressed in the developing diaphragms of leaf sheaths (Fig. 4f).

*OsGH3–4*, a member of the *GH3* family involved in auxin conjugation, was placed in Cluster 7 and exhibited elevated expression levels mainly in the leaf sheath and blade-sheath boundary region. Expression of *OsGH3–4* was detected in both the central domain of the SAM and tissues adaxially adjacent to vascular bundles in the leaf sheath and midrib, where bundle sheath extension cells differentiate (Fig. 4g, h).

**Fig. 5:**
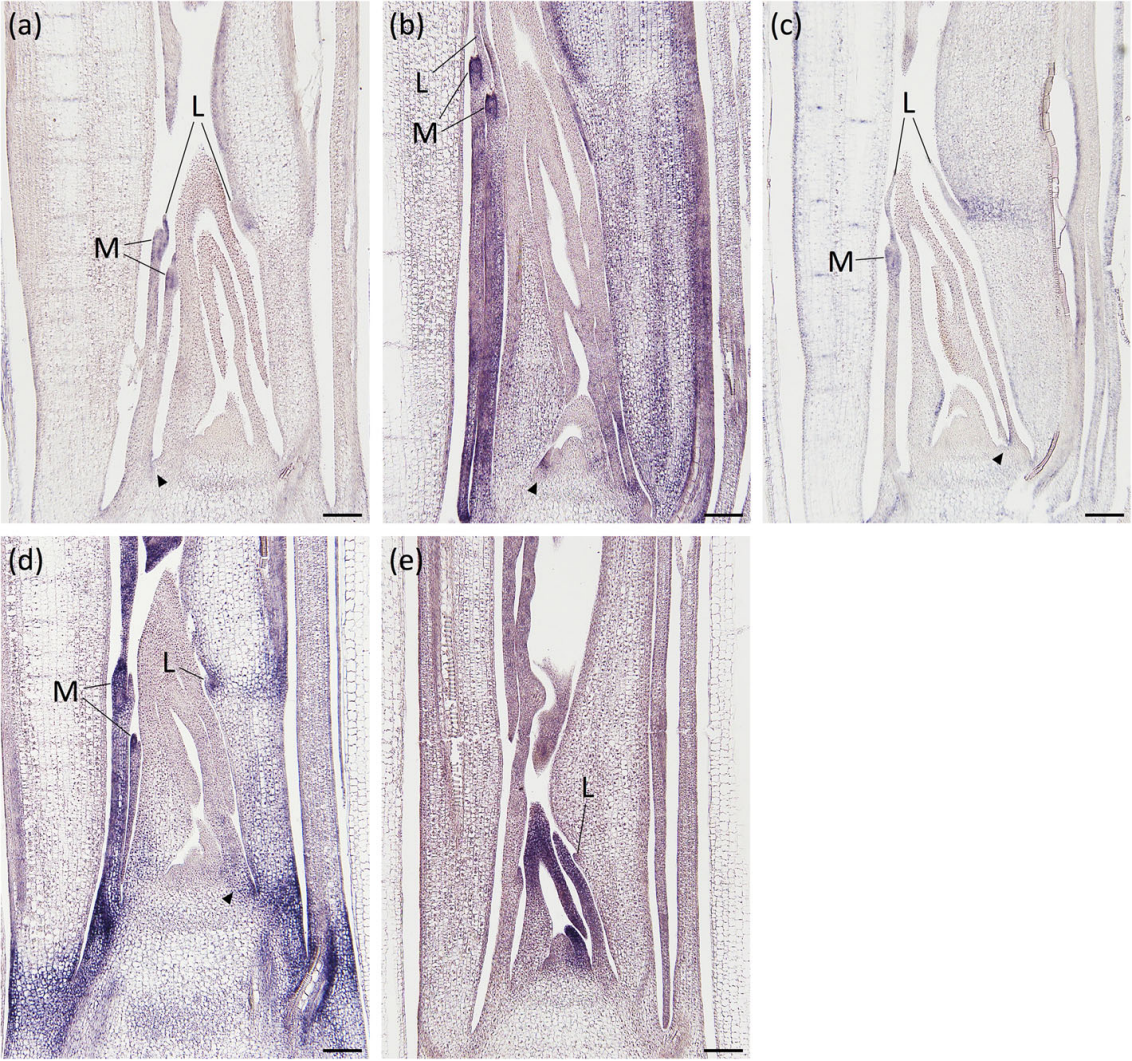
Spatial expression patterns of four *OsARF*s and the accumulation pattern of mature *miR167*. The expression patterns of **a** *OsARF6,* **b** *OsARF12,* **c** *OsARF17*, **d** *OsARF25*, and **e** mature *miR167* around the shoot apex in longitudinal sections. Arrowheads in (**a**)–(**d**) indicate expression in the basal part of the leaf margin. L, ligule; M, marginal part of the blade-sheath boundary. Scale bars: 100 μm

Additionally, Cluster 7 contained four *OsARF* paralogs belonging to the *ARF6/8* subfamily [46]. These genes, *OsARF6/12/17/25,* were expressed at the basal part of the leaf primordium around stage P3, with especially high expression levels at the margin (Fig. 5a–d, arrowheads). Moreover, these four *OsARF*s were also expressed in the developing ligule and marginal parts of the blade-sheath boundary region that were expected to differentiate into auricles (Fig. 5a–d). In addition to these common expression patterns among the four *OsARF*s, the characteristic expression of each gene was also observed. *OsARF6* was expressed in the epidermis of the basal region of P4 leaf blades (Fig. 5a), whereas *OsARF12* was expressed throughout the sheath and basal region of the blades (Fig. 5b). *OsARF17* expression was detected in the adaxial epidermis at the blade-sheath boundary at stage P3 and in the lamina joint of P4 leaves (Fig. 5c), whereas *OsARF25* was strongly expressed in the lamina joint of P4 leaves and at the base of sheaths in stages P4 and P5 (Fig. 5d).

In addition, *ARF6/8* orthologs including the four *OsARF*s listed above are known targets of *miR167*, which is an evolutionarily conserved microRNA in seed plants [46]. To clarify the relationships between the expression patterns of the four *OsARF*s and *miR167*, the accumulation patterns of mature *miR167* in the shoot apex were examined through in situ hybridization using a probe containing BNA^NC^, which is a bridged nucleic acid derivative of a locked nucleic acid (Fig. 5e). Signals specific to mature *miR167* were detected at higher levels in the distal part and lower levels in the basal part, indicating that the accumulation of *miR167* and the expression of the four identified *OsARF*s were largely exclusive.

In addition to the genes described above, we explored a number of genes with localized expression during leaf development based on the list obtained through K-means clustering (Supplemental Figure 8; Supplemental Table 2). Therefore, our strategy of using K-means clustering facilitated the selection of genes that were differentially expressed among stages or tissues. In addition, the genes identified here should be further analyzed to uncover their functions in rice leaf development.

## Discussion

Leaf primordia, which originate from the flank of the SAM, undergo various developmental processes from differentiation to maturation. Leaf primordia of grasses develop distinct regions along the longitudinal axis, and each region differentiates into a morphologically unique structure. Tissue differentiation in grass leaves during development proceeds in a basipetal direction, which has been extensively studied using the maize developing leaf blade as a model [16, 47–54]. These studies revealed that basipetal tissue differentiation is accompanied by dynamic changes in mRNA, proteins, and various metabolites. However, the resolution of these studies is limited spatially and temporally, as only parts of a single developing leaf blade at a specific stage were used, whereas other stages and tissues were not examined. Thus, our study is unique in terms of providing genome-wide expression profiles both across developmental stages and distinct tissues along the longitudinal axis.

In this study, the PCA results suggest that the leaf transcriptome dynamically changes among stages of tissue differentiation, as shown in the space defined by mPC1 and mPC2 (Fig. 2b). On the other hand, the difference between the leaf blade and sheath, which was represented by mPC3, accounted for less transcriptomic variation among samples than did mPC1 or mPC2 (Fig. 2c), suggesting that organ identity has a smaller effect on the leaf transcriptome than tissue differentiation. In particular, marked transcriptomic changes were observed in the P4 leaf along its longitudinal axis (Fig. 2b). The transcriptomes of the proximal parts, P4S and P4Bb, resembled that of the leaf primordium in early stages, such as P3. On the other hand, the distal part (P4Ba) exhibited transcriptomic similarities to leaf parts at later stages, such as P5 and P6. Thus, our results suggest that P4 is the stage at which tissue differentiation proceeds in the basipetal direction along the whole leaf and dynamic transcriptome changes occur.

Along the longitudinal axis of developing grass leaves, it has been suggested that cell proliferation and elongation occur actively in the basal and middle regions [55] and that photosynthetic activity and related gene expression is high in the apical region [16, 53]. Our cluster and GO analyses revealed that the genes in Clusters 2, 9, and 12 may be involved in cell proliferation, cell elongation, and photosynthesis, respectively (Fig. 3; Table 2). The expression patterns of these clusters in various parts of the P4 leaf are consistent with the previous findings; namely, different developmental events occur in a single leaf primordium along its longitudinal axis [16, 53, 55]. Thus, P4 is the stage wherein drastic developmental reprograming occurs in rice. Moreover, acquisition of photosynthetic competence is initiated between the P3 and P4 stages [24], which is in accordance with our finding that genes in Cluster 12 were weakly expressed at stage P3 and strongly expressed at stage P4 (Fig. 3; Table 2). In addition to these clusters, we identified Cluster 7 genes as being highly expressed in the sheath and blade-sheath boundary regions (Fig. 3; Table 2). *OsBOP* genes, which are regulators of leaf sheath identity, were included in this cluster and highly expressed at stage P3, suggesting that a number of upstream or downstream genes exhibit polarized expression patterns along the longitudinal axis from an early stage of leaf development.

The rice leaf has distinct morphological features that are not present in *Arabidopsis*. The development of these features is likely controlled in part by genetic mechanisms unique to grasses. Previous studies have identified several genetic factors in the morphogenesis of characteristic organs in grass leaves. *LIGULELESS1* (*LG1*) is required for the development of structures at the boundary between the blade and sheath [2–4]. *DROOPING LEAF* is important for the development of the midrib [56, 57]. However, most of the genetic factors and their networks that underpin grass leaf morphogenesis remain unknown.

In general, to explore novel genes that play roles in the developmental process of interest, an efficient screening method is required. Expression profiling is one practical method of gene discovery. Transcriptome analysis and in situ hybridization are representative methods of expression analysis with different advantages. Transcriptome analysis can provide global expression profiles at the genome-wide scale, whereas in situ hybridization can reveal the spatial expression pattern of a gene at the tissue and cellular levels. By combining these methods, many studies have attempted to identify novel genes that function in a developmental process of interest [15, 22, 58–65].

In this study, we identified a number of genes with localized expression patterns that may be associated with morphological features unique to rice and grasses. For example, *Os02g0172800* was expressed in immature leaf primordia at higher levels in the abaxial side of the basal part of the leaf primordia (Fig. 4a, b). This expression pattern is similar to that of *MKB3*, which is a positive regulator of cell proliferation in leaf primordia [11]. Furthermore, *Os02g0172800* is a co-ortholog of *ORESARA15*, which reportedly promotes cell proliferation through a genetic pathway mediated by *AN3*, an ortholog of *MKB3* [45]. It has been suggested that the formation of the leaf sheath requires a decreasing gradient of cell proliferation from the abaxial side of the leaf primordium to the adaxial side, which may be associated with the expression pattern of *MKB3* [11]. Thus, *Os02g0172800* might be also involved in cell proliferation in leaf primordia, and its expression pattern might reflect the unique cell proliferation pattern during the development of the leaf sheath.

Expression of *OsbHLH80* was detected in the developing ligule (Fig. 4c). Ligule development was found to be disrupted by dysfunction of brassinosteroid (BR) signaling [66]. *OsBC1*, a paralog of *OsbHLH80*, is considered to regulate lamina-joint bending in response to BRs [67]. Thus, characterizing the roles of *OsbHLH80* in ligule development in association with BR signaling is a worthwhile topic for future research.

*OsGH3–4* was expressed in tissues adaxially adjacent to vascular bundles in the leaf sheath and midrib, where bundle sheath extension cell differentiation occurs (Fig. 4g, h). In addition, tissue-specific expression of *OsbHLH166* and *Os05g0363500* were observed in the presumptive region of lysigenous aerenchyma and developing diaphragms, respectively (Fig. 4d–f). During the development of the leaf sheath and midrib, a group of parenchyma cells collapse to form lysigenous aerenchyma, whereas parenchyma cells adaxially adjacent to the vascular bundles remain intact and differentiate into bundle sheath extension cells. Additionally, some parenchyma cells transform into stellate parenchyma cells in diaphragms that vertically separate the lysigenous aerenchyma [68]. Although these structures are well-developed in the leaves of rice and some wetland plants, the genetic networks regulating the development of these structures remain unknown. It has been demonstrated that the formation of constitutive aerenchyma in rice roots is regulated by auxin signaling [69]. Thus, *OsGH3–4* may play an important role in the patterning of bundle sheath extension cells through the modulation of the spatial patterns of auxin accumulation in leaf primordia. Functional analysis of *OsbHLH166* and *Os05g0363500* would provide insights into the developmental programs underlying the formation of aerenchyma and elaboration of the unique cell shape of stellate parenchyma cells forming diaphragms.

*OsARF6/12/17/25* exhibited polarized expression along the longitudinal axis and was expressed in developing ligules as well as the marginal parts of the blade-sheath boundary, where auricle differentiation occurs (Fig. 5a–d). Moreover, *OsARF17* exhibited a unique expression pattern in the adaxial epidermis of the blade-sheath boundary in leaf primordia (Fig. 5c). Similarly, *OsLG1* exhibited localized expression in marginal parts of the blade-sheath boundary (Supplemental Figure 9). Recent research suggested that a member of the *ARF6/8* subfamily acts downstream of the *OsLG1* ortholog in wheat [4]. Thus, these genes may be important for the development of organs in the blade-sheath boundary region. Furthermore, *OsARF25* exhibited strong expression in the leaf-sheath pulvinus, which is a gravisensitive tissue in the leaf base that is involved in shoot bending in grasses [70] (Fig. 5d). This expression pattern suggests that *OsARF25* may play an important role in the bending capability of shoots at the leaf-sheath pulvinus in response to gravity. In addition to the leaf-sheath pulvinus, *OsARF25* was strongly expressed in the lamina joint (Fig. 5d). Several genes controlling shoot bending were previously reported to be expressed in both the lamina joint and leaf-sheath pulvinus [71–73]. These gene expression patterns suggest that the lamina joint and leaf-sheath pulvinus use similar genetic programs to achieve bending.

Post-transcriptional regulation by *miR167* has been reported to be required for attaining the correct spatial expression patterns of *ARF6/8* during reproductive development in *Arabidopsis* [74]. Our analyses revealed mutually exclusive expression of *miR167* and *OsARF6/12/17/25* along the longitudinal axis of leaf primordia. This result indicates that *miR167* post-transcriptionally downregulates *OsARF6/12/17/25* expression in the distal part of the leaf primordium (Fig. 5). A similar accumulation pattern for mature *miR167* was reported in *Arabidopsis* cotyledons [75]. Thus, post-transcriptional regulation of *ARF6/8* orthologs by *miR167* in the distal part of the leaf primordium may be evolutionally conserved between rice and *Arabidopsis*.

Our microarray analysis had some limitations in resolution despite the sampling of extensive leaf stages and regions. For example, the sample SA contained the SAM, P1 and P2 leaf primordia, and immature stem tissues below the shoot apex, which makes it impossible to distinguish differences in expression profiles among these tissues (Fig. 1d). To improve our transcriptomic resolution, the separation of small tissues through laser microdissection, as performed in maize transcriptome studies on different domains of the SAM [15, 22], may facilitate more detailed gene expression profiling at the early stages of rice leaf development. In addition, it is possible that gene expression would change as plants grow mature. As a preliminary test for the possibility, we analyzed temporal expression profiles of six genes in shoot samples from the second to the forth leaf stage by real-time RT-PCR analysis (Supplemental Figure 3). Four genes are equally expressed among the three developmental stages (Supplemental Figure 3a to d), whereas *OsPsbR3* showed a decreasing trend (Supplemental Figure 3e). *OsLOX2;2* showed extremely low expression in all the developmental stages, presumably because the shoot tissue used in this experiment did not contain the distal part of leaf blade where this gene is highly expressed (Supplemental Figure 3f). The result indicated that expression of some genes could change even within the short growth period. To clarify expression profiles of genes throughout the developmental stages of plant life cycle, a large-scale transcriptome analysis is required in future studies. Moreover, the 44 K microarray platform used in this study does not cover all transcripts in the rice genome. RNA sequencing analysis is required to obtain the expression profiles of all transcripts and identify a greater number of novel genes related to leaf development. However, our expression data are indispensable for elucidating the global transcriptome and true nature of individual gene expression levels in rice due to their compatibility with various datasets in the expression profile database RiceXPro (http:// ricexpro.dna.affrc.go.jp) [26, 27].

## Conclusions

We identified a large number of genes that exhibit localized and unique expression patterns during rice leaf development (Figs. 4 and 5; Supplemental Figure 8; Supplemental Table 2). Due to the recent development of CRISPR/Cas9 technology, any genes in the rice genome can be easily knocked out [76]. Thus, reverse genetics strategies to reveal gene function can be applied to the genes identified in this study. Our findings will provide the foundation for future research on the development of grass leaves and contribute to the elucidation of genetic programs unique to grasses.

## Methods

### Plant materials and growth conditions

Rice (*Oryza sativa L.* ssp. japonica cv. Nipponbare) seeds were obtained from the National Agriculture and Food Research Organization. They were sown in germination boxes, and the seedlings were grown in a growth chamber (14-h light period at 30 °C and 10-h dark period at 25 °C). At 14–15 days after germination, seedlings in which the tip of the fifth leaf had just emerged from the fourth leaf were collected. The seedlings were dissected under a dissecting microscope to separate tissues at different developmental stages and locations along the longitudinal axis of the leaves. The dissected tissues were used for RNA extraction of microarray analysis.

### RNA extraction and microarray analysis

Total RNA was extracted from the collected samples using a RNeasy Mini Kit (Qiagen, Hilden, Germany), and labeling was performed using Quick Amp Labeling Kit, One-Color (Agilent Technologies) in the presence of cyanine-3 (Cy3)-CTP according to the manufacturer’s protocol. The resulting Cy3-labeled cRNA was purified using a RNeasy Mini Kit (Qiagen, Hilden, Germany). A total of 1000 ng Cy3-labeled cRNA was fragmented and hybridized onto a slide of the rice 4 × 44 K microarray RAP-DB (G2519F#15241; Agilent Technologies). After washing, the slides were scanned on an Agilent G2505B DNA microarray scanner, and background correction of the raw Cy3 signals was performed using Feature Extraction 10.5.1.1 software (Agilent Technologies).

### Statistical analysis

The processed raw signal intensities of all probes were transformed to log_2_ scale, and normalization procedures were performed, including 75th-percentile normalization for inter-array comparison and baseline correlation of each probe to the median value of all the samples, using GeneSpring GX12 software (Agilent Technologies). In this study, we used 31,996 probes corresponding to 24,022 loci, which had raw signal intensities > 50 in at least one of the 36 microarray datasets. A density plot of probes for each sample was drawn using ggridges package in R [77]. Pearson correlation analysis was performed on all samples using the ggcorrplot package in R [78]. PCA based on the variance-covariance matrix was first performed on all samples using the prcomp function in R. Subsequently, PCA was applied again to all samples except for P4Bm, using the PC1, PC2, and PC3 scores as variables to compute modified principal components (mPC1, mPC2, and mPC3). The distribution of the samples in the space defined by mPC1 and mPC2 was approximated with a quadratic formula using the nls function in R. One-way ANOVA with Benjamini and Hochberg FDR correction was applied to detect the probes that were differentially expressed among samples (FDR-adjusted *p*-value = 0.001) using GeneSpring GX12. The extracted probes were classified into 28 clusters through K-means cluster analysis based on Euclidean distance using the cclust package in R [79]. GO enrichment analysis was performed on the genes in each cluster using a tool based on a rice gene coexpression database, RiceFREND [80]. GO terms corresponding to fewer than five genes were discarded. As GO terms are organized in a hierarchical structure, only the child terms were retained. Transcription factor and transcriptional regulator genes were extracted from each cluster using the list in the Plant Transcription Factor Database [81]. In addition, the enrichment of each gene family in each cluster was statistically tested by comparing the number of family members in each cluster with that obtained from the rice 44 K microarray platform, using one-sided Fisher’s exact tests with Benjamini and Hochberg FDR correction with the fisher.test and p.adjust functions in R. The expression profiles shown in Supplemental Figure 3, 5 and Fig. 3, and the heatmaps in Supplemental Figsure 6 and 7 were drawn using the ggplot2 package in R [82].

### Gene annotation used for analyses

The gene annotation used in this study was obtained from RAP-DB [29, 30]. The list of genes associated with the biosynthesis, catabolism, and signaling of six phytohormones was derived from Hirano et al. (2008) [83]. For the extraction of transcription factor and transcriptional regulator genes from the rice genome, the list from the Plant Transcription Factor Database [81] was used.

### In situ hybridization

Rice (*O. sativa* ssp. japonica cv. Nipponbare) seeds were obtained from the National Agriculture and Food Research Organization. They were sown in soil, and the seedlings were grown in a growth chamber (14-h light period at 30 °C and 10-h dark period at 25 °C). At 14–15 days after germination, the shoot apex of each four-leaf stage seedling was dissected and fixed in 4% paraformaldehyde in 0.1 M sodium phosphate buffer for 24 h at 4 °C, and then dehydrated in a graded ethanol series. The ethanol was replaced with Histo-Clear (National Diagnostics, Atlanta, GA), and the samples were embedded in Paraplast Plus (Leica, Wetzlar, Germany). Paraffin sections (thickness, 8 μm) were placed on microscope slides and coated with 3-aminopropyl triethoxysilane (Matsunami Glass, Osaka, Japan). To generate probes for 30 genes (see Supplemental Table 3), the corresponding full-length cDNA clones were obtained from NIAS Genebank [84] and used as templates. For 20 genes, cDNA fragments were amplified using PCR and cloned into the pCR Blunt II TOPO vector (Invitrogen, Carlsbad, CA) using primers specific to each gene (Supplemental Table 3). The cDNA was amplified via PCR or digested with restriction enzymes and then transcribed using digoxigenin-labeled antisense riboprobes using a MAXIscript In Vitro Transcription kit (Life Technologies, Carlsbad, CA) with digoxigenin-11-UTP (Roche, Basel, Switzerland), or using T7, SP6 (Takara, Shiga, Japan), or T3 (Roche) RNA polymerase with DIG-RNA labeling mix (Roche). For detecting mature *miR167*, a 3′-end digoxigenin-labeled probe with the sequence 5′-AgAtCaTgCtGgCaGcTtCa-3′ was used, where uppercase and lowercase letters represent BNA^NC^[NMe] and DNA, respectively. In situ hybridization and immunological detection of the hybridization signals were performed as described by Kouchi and Hata (1993) [85].

### Real-time RT-PCR analysis

For verification of expression profiles, the same RNA samples from the four parts of P4 stage: P4S, P4Bb, P4Bm and P4Ba (Table 1) as those used for microarray were examined. For examination of temporal expression profiles, RNA samples from 1 cm of the most basal part of rice shoots at the second, third and fourth leaf stage were used. RNA was treated with TURBO DNA-free™ Kit (Invitrogen, Carlsbad, CA), and cDNA was synthesized using the High Capacity cDNA Reverse Transcription Kit (Applied Biosystems, Foster City, CA). Quantitative RT- PCR was performed with the StepOne™ Real-Time PCR System (Life Technologies, Carlsbad, CA) using the TaqMan Fast Universal PCR Master Mix and FAM-labeled TaqMan probes for each gene. *OsRAD6* (*Os03g0791800*) was used as an internal standard [31]. Three technical replicates were performed for each sample. The primers and TaqMan probes for each gene are listed in Supplemental Table S4.

## Supplementary Information

**Additional file 1: Supplemental Figure S1.** Distribution of the normalized intensity values of probes for each sample. **Supplemental Figure S2.** Heatmap showing Pearson correlation coefficient (PCC) values representing the relationships between samples. **Supplemental Figure S3.** Verification of expression profiles of six genes by real-time RT-PCR analysis. **Supplemental Figure S4.** Principal component analysis score plot of samples based on the original principal components. **Supplemental Figure S5.** Expression patterns of 28 clusters obtained through K-means analysis. **Supplemental Figure S6.** Heatmap of Gene Ontology (GO) terms overrepresented in each cluster. **Supplemental Figure S7.** Numbers of transcription factors and transcriptional regulators in each cluster.

**Additional file 2: Supplemental Figure S8.** In situ hybridization of genes showing localized expression during leaf development. **Supplemental Table 2.** Annotation of genes in Fig. 4, 5. **Supplemental Figure S9.** Spatial expression pattern of *LIGULELESS1*.

**Additional file 3:.** Three-dimensional model of Supplemental Figure 4. A three-dimensional model of PCA score plot of samples based on the original principal components. The proportions of the total variance explained by PC1, PC2, and PC3 are shown in parentheses. Samples collected at the same stage are shown in the same color. Samples with different tissue identities are indicated by different symbols: shoot apex, square; P3 leaf, circle; blade, triangle; blade-sheath boundary, diamond; sheath, inverted triangle. Red arrows represent the directions of the modified principal components (mPC1, mPC2, and mPC3) shown in Fig. 2.

**Additional file 4:.** Three-dimensional model of Fig. 2. A three-dimensional model of PCA score plot of samples based on the modified principal components. The proportions of the total variance explained by PC1, PC2, and PC3 are shown in parentheses. Samples collected at the same stage are shown in the same color. Samples with different tissue identities are indicated by different symbols: shoot apex, square; P3 leaf, circle; blade, triangle; blade-sheath boundary, diamond; sheath, inverted triangle. Red arrows represent the directions of the original principal components (PC1, PC2, and PC3) shown in Supplemental Figure 4.

**Additional file 5: Supplemental Table S1**. The list of genes categorized into 28 clusters by K-means analysis.

**Additional file 6: Supplemental Table S3**. The list of full-length cDNA clones and cloning primers used for preparation of probes for in situ hybridization.

**Additional file 7: Supplemental Table S4**. The list of primers and TaqMan probes used for real-time RT-PCR analysis.

## Data Availability

The microarray datasets generated and analyzed during the current study are available in the Gene Expression Omnibus (GEO) repository through accession number GSE159047 [https://www.ncbi.nlm.nih.gov/geo/query/acc.cgi?acc=GSE159047]. The list of transcription factor and transcriptional regulator genes are available in the Plant Transcription Factor Database [http://plntfdb.bio.uni-potsdam.de/v3.0/].

## Abbreviations

*SPL*: *SQUAMOSA PROMOTER BINDING-LIKE*
SAM: Shoot apical meristem
*GRF*: *GROWTH-REGULATING FACTORS*
*GIF*: *GRF-INTERACTING FACTORS*
Pn: Plastchron number
PCA: Principal component analysis
ANOVA: Analysis of variance
FDR: False discovery rate
GO: Gene ontology
*PIF*: *Phytochrome-interacting bHLH factors*
BNA^NC^: Bridged nucleic acid
*LG1*: *LIGULELESS1*
BR: Brassinosteroid
RT-PCR: Reverse transcription PCR
GEO: Gene Expression Omnibus
