# Supplementary material for: Genome-wide analysis of spatiotemporal expression patterns during rice leaf development
Source: BMC Genomics. 2021 Mar 9;22:169. doi: 10.1186/s12864-021-07494-5 (PMC7941727; doi:10.1186/s12864-021-07494-5)
Supplement: Supplementary file 1 — Additional file 1: Supplemental Figure S1. Distribution of the normalized intensity values of probes for each sample. Supplemental Figure S2. Heatmap showing Pearson correlation coefficient (PCC) values representing the relationships between samples. Supplemental Figure S3. Verification of expression profiles of six genes by real-time RT-PCR analysis. Supplemental Figure S4. Principal component analysis score plot of samples based on the original principal components. Supplemental Figure S5. Expression patterns of 28 clusters obtained through K-means analysis. Supplemental Figure S6. Heatmap of Gene Ontology (GO) terms overrepresented in each cluster. Supplemental Figure S7. Numbers of transcription factors and transcriptional regulators in each cluster. [file 12864_2021_7494_MOESM1_ESM.docx]

Supplemental Fig. 1. Distribution of the normalized intensity values of probes for each sample.

The curve for each sample represents a density plot of 31996 probes expressed at least in one sample. Ends of each curve correspond to the maximum and minimum values within each sample.

Supplemental Fig. 2. Heatmap showing Pearson correlation coefficient (PCC) values representing the relationships between samples.

The numbers in cells indicate PCC values between samples.

High PCC values are shown in red and low PCC values are shown in blue.

Supplemental Fig. 3. Verification of expression profiles of six genes by real-time RT-PCR analysis.

Expression profile of (a) *Os09g0466100 (OsCYCD4;1)*, (b) *Os01g0948900 (OsBOP1)*, (c) *Os03g0368900 (prx45)*, (d) *Os03g0170900 (OsSUT1)*, (e) *Os08g0200300 (OsPsbR3)*, (f) *Os12g0559200 (OsLOX2;2)*.

For each gene, the left panel shows the expression pattern by microarray analysis. The center and the right panels show the relative expression by real-time RT-PCR analysis, which were normalized by that of *OsRAD6*. Bars indicate s.d. from three technical replicates.

S2, second leaf stage shoot; S3, third leaf stage shoot; S4, fourth leaf stage shoot; ND, not detected.

Supplemental Fig. 4. Principal component analysis score plot of samples based on the original principal components.

(a) The space defined by PC1, PC2, and PC3. Red arrows in panel (a) represent the directions of the modified principal components (mPC1, mPC2, and mPC3) shown in Fig. 2. A three-dimensional model allowing interactive rotation is available in additional file 3.

(b) The space defined by PC1 and PC2.

(c) The space defined by PC1 and PC3.

The proportions of the total variance explained by PC1, PC2, and PC3 are shown in parentheses. Samples collected at the same stage are shown in the same color. Samples with different tissue identities are indicated by different symbols: shoot apex, square; P3 leaf, circle; blade, triangle; blade-sheath boundary, diamond; sheath, inverted triangle.


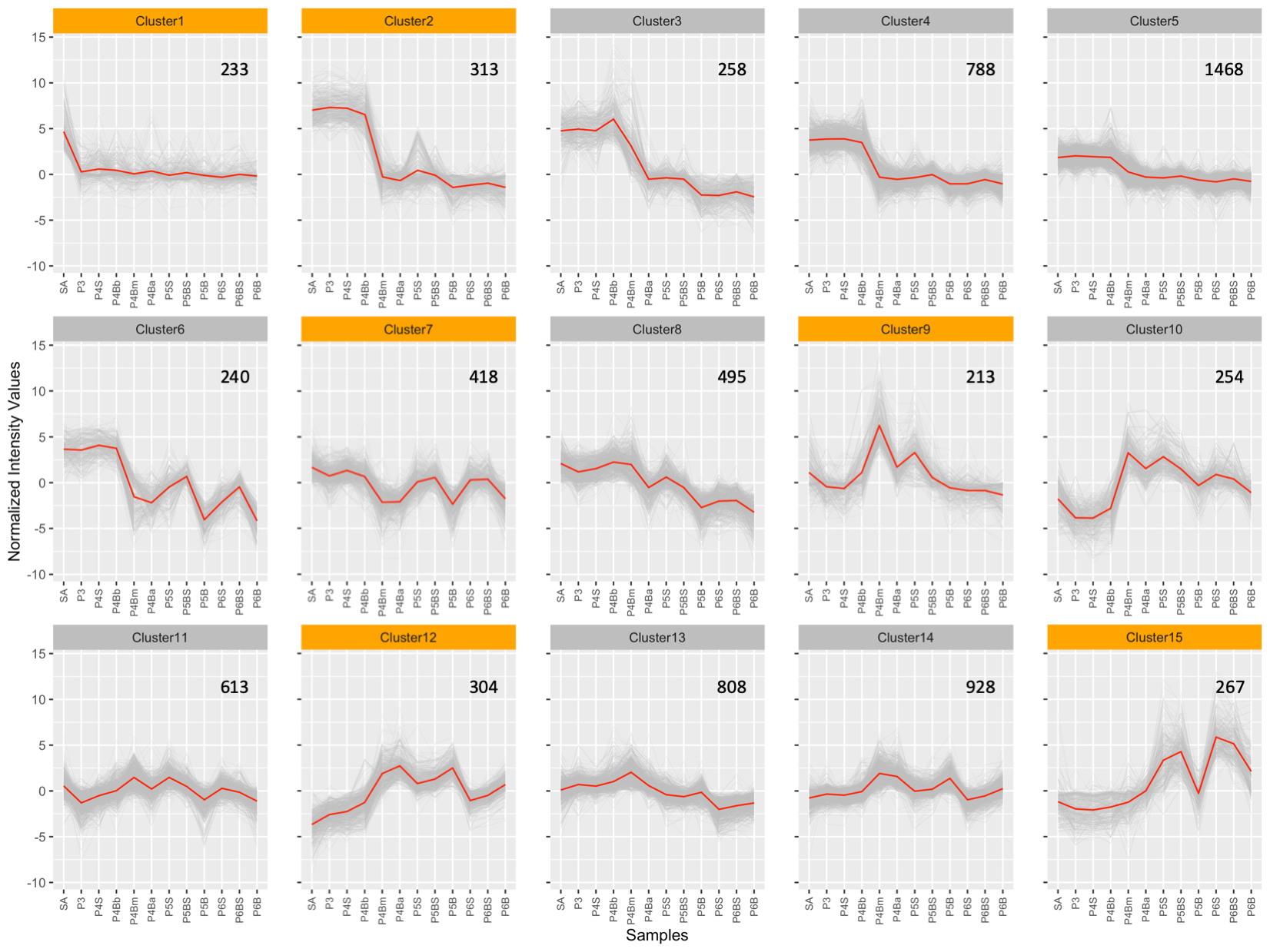


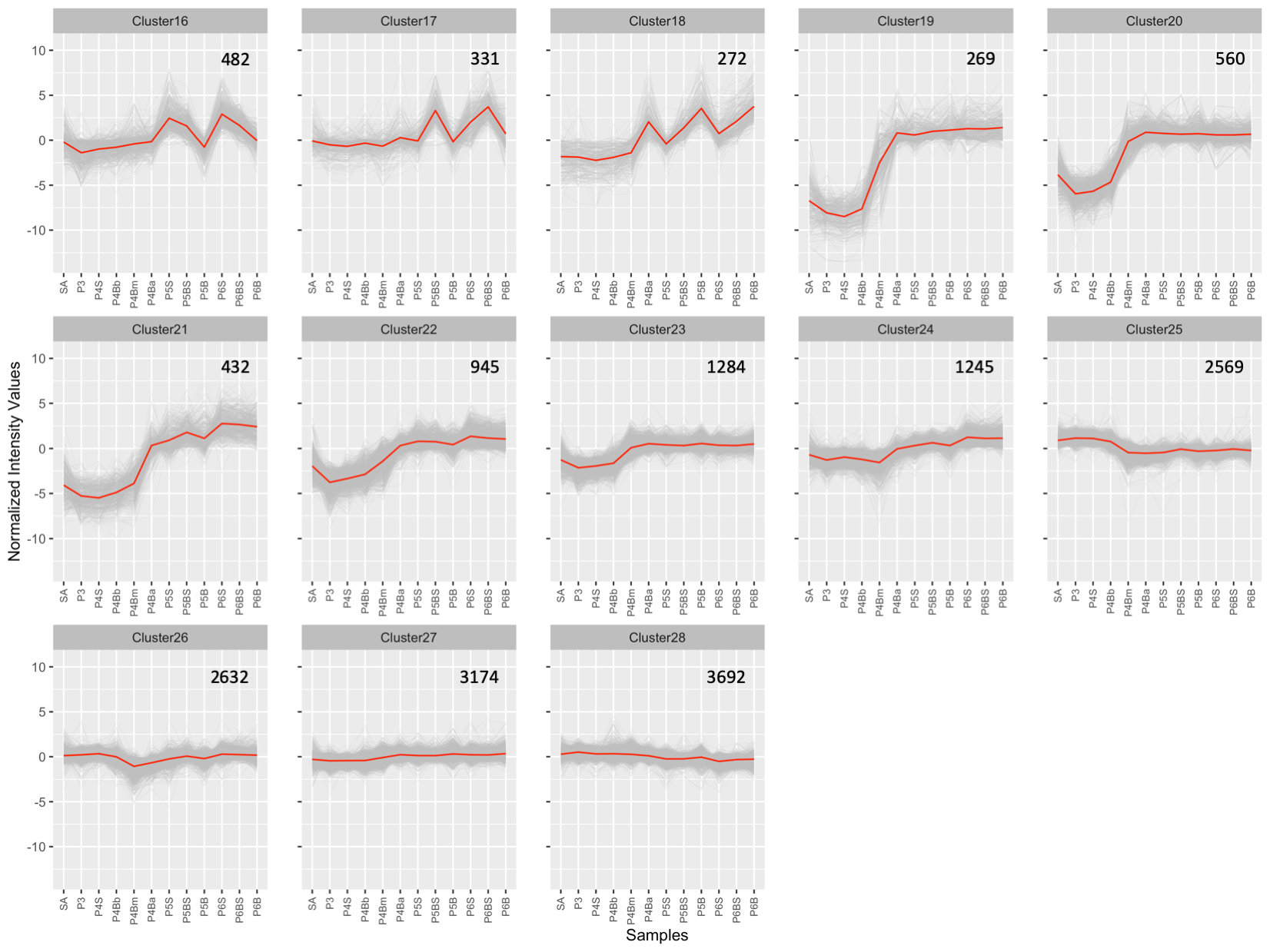


Supplemental Fig. 5. Expression patterns of 28 clusters obtained through K-means analysis.

Grey lines indicate the expression profiles of the probes in each cluster.

Red lines indicate the mean of all probes in each cluster.

Three biological replicates are summarized by median.

The number of genes in each cluster is indicated in the upper right.

The six clusters shown in Fig. 3 are highlighted in orange.

The list of genes in each cluster is available in Supplemental Table 1.

Supplemental Fig. 6. Heatmap of Gene Ontology (GO) terms overrepresented in each cluster.

The columns represent 28 clusters obtained through K-means analysis, and rows represent GO terms. The cell color represents the p-value associated with the corresponding GO term, and the numbers in cells indicate the number of genes associated with the GO term in the corresponding cluster. GO terms described in the main text are outlined with black boxes.

Supplemental Fig. 7. Numbers of transcription factors and transcriptional regulators in each cluster.

The columns represent 28 clusters obtained through K-means analysis, and rows represent gene families with the numbers of members found in the rice 44K microarray platform. The numbers in cells indicate the number of gene family members in each cluster. Cell color indicates the p-value for the enrichment of gene families in the cluster. Gene families described in the main text are outlined with black boxes.
