# Supplementary material for: Genome-wide analysis of spatiotemporal expression patterns during rice leaf development
Source: BMC Genomics. 2021 Mar 9;22:169. doi: 10.1186/s12864-021-07494-5 (PMC7941727; doi:10.1186/s12864-021-07494-5)
Supplement: Supplementary file 2 — Additional file 2: Supplemental Figure S8. In situ hybridization of genes showing localized expression during leaf development. Supplemental Table 2. Annotation of genes in Fig. 4, 5. Supplemental Figure S9. Spatial expression pattern of LIGULELESS1. [file 12864_2021_7494_MOESM2_ESM.docx]

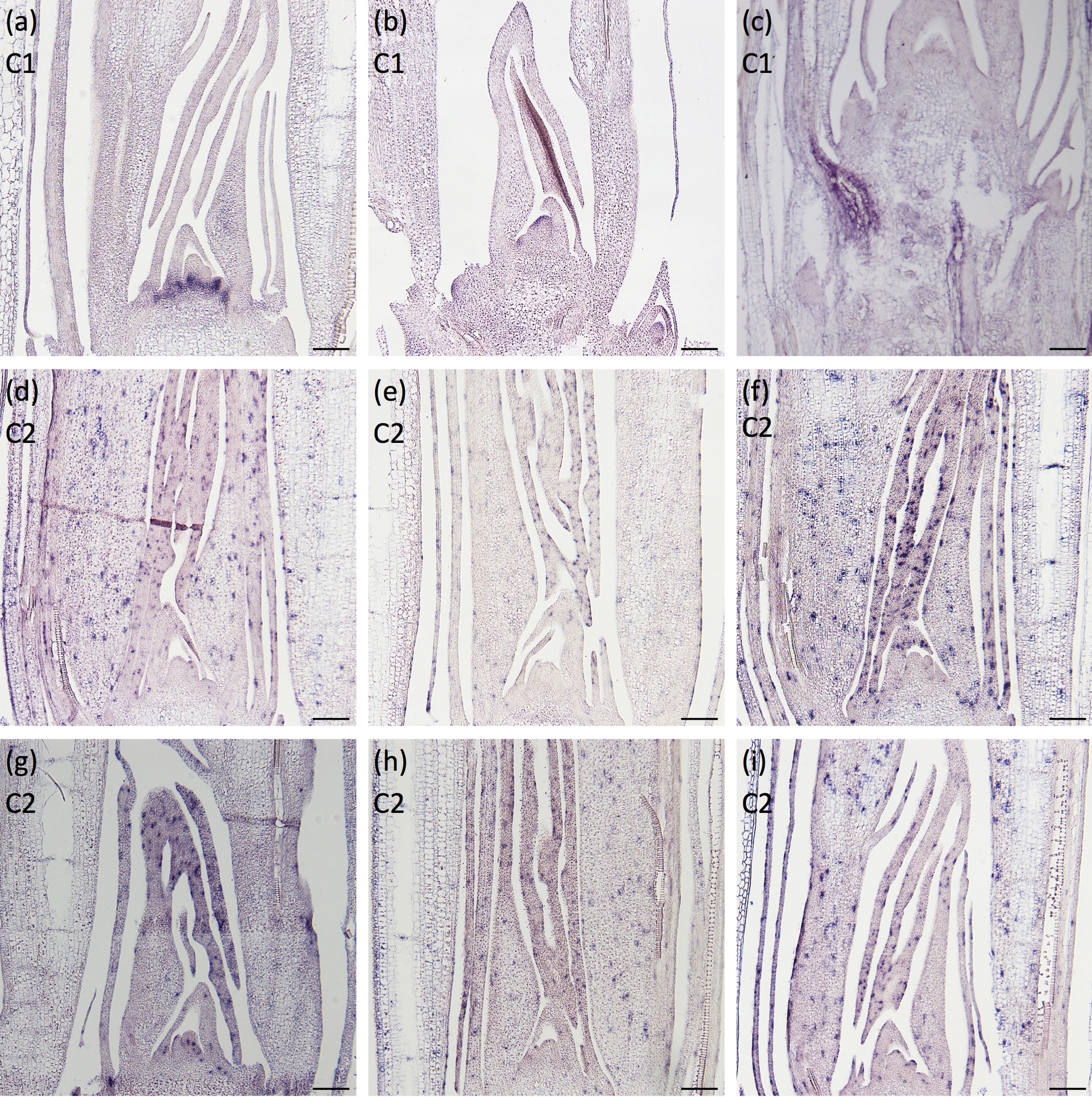


Supplemental Fig. 8. *In situ* hybridization of genes showing localized expression during leaf development.

The expression patterns of (a) *Os05g0223200*, (b) *Os05g0153200*, (c) *Os10g0579400*, (d) *Os01g0176500*, (e) *Os01g0765500*, (f) *Os02g0258200*, (g) *Os03g0184500*, (h) *Os04g0228100*, and (i) *Os09g0556800* around the shoot apex in longitudinal sections.

The cluster number to which each gene belongs is indicated in the upper left.

See Supplemental Table 2 for annotation of the genes.

Scale bars: 100 μm.


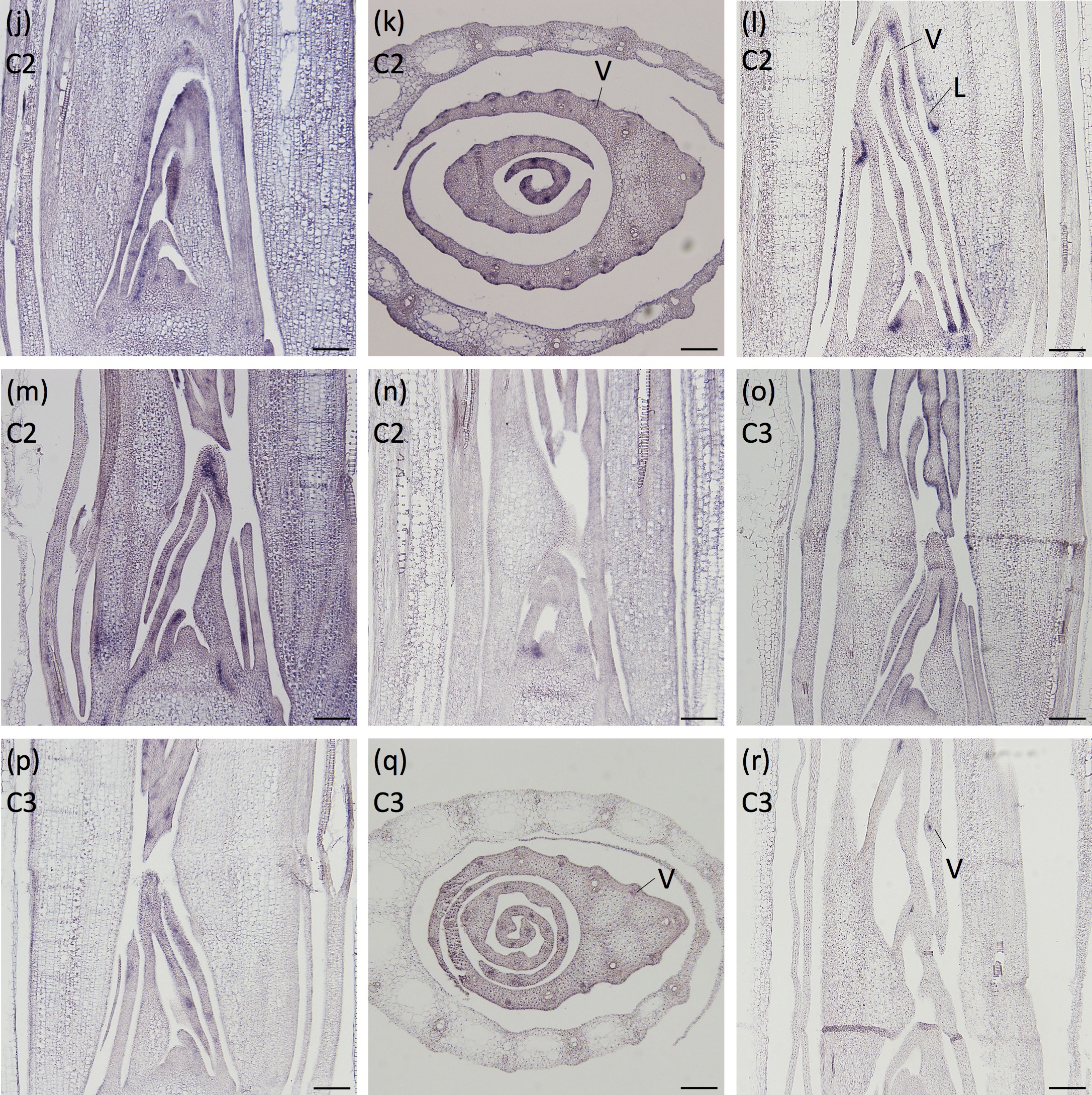


Supplemental Fig. 8. (Continued)

The expression patterns of (j, k) *Os11g0149100*, (l) *Os04g0664400*, (m) *Os03g0244600*, (n) *Os03g0680700*, (o) *Os03g0164300*, (p, q) *Os06g0474500*, and (r) *Os02g0528100* around the shoot apex.

(j), (l) to (p), and (r) are longitudinal sections of the shoot apices, while (k) and (q) are transverse sections of the shoot apices.

The cluster number to which each gene belongs is indicated in the upper left.

See Supplemental Table 2 for annotation of the genes.

Scale bars: 100 μm.


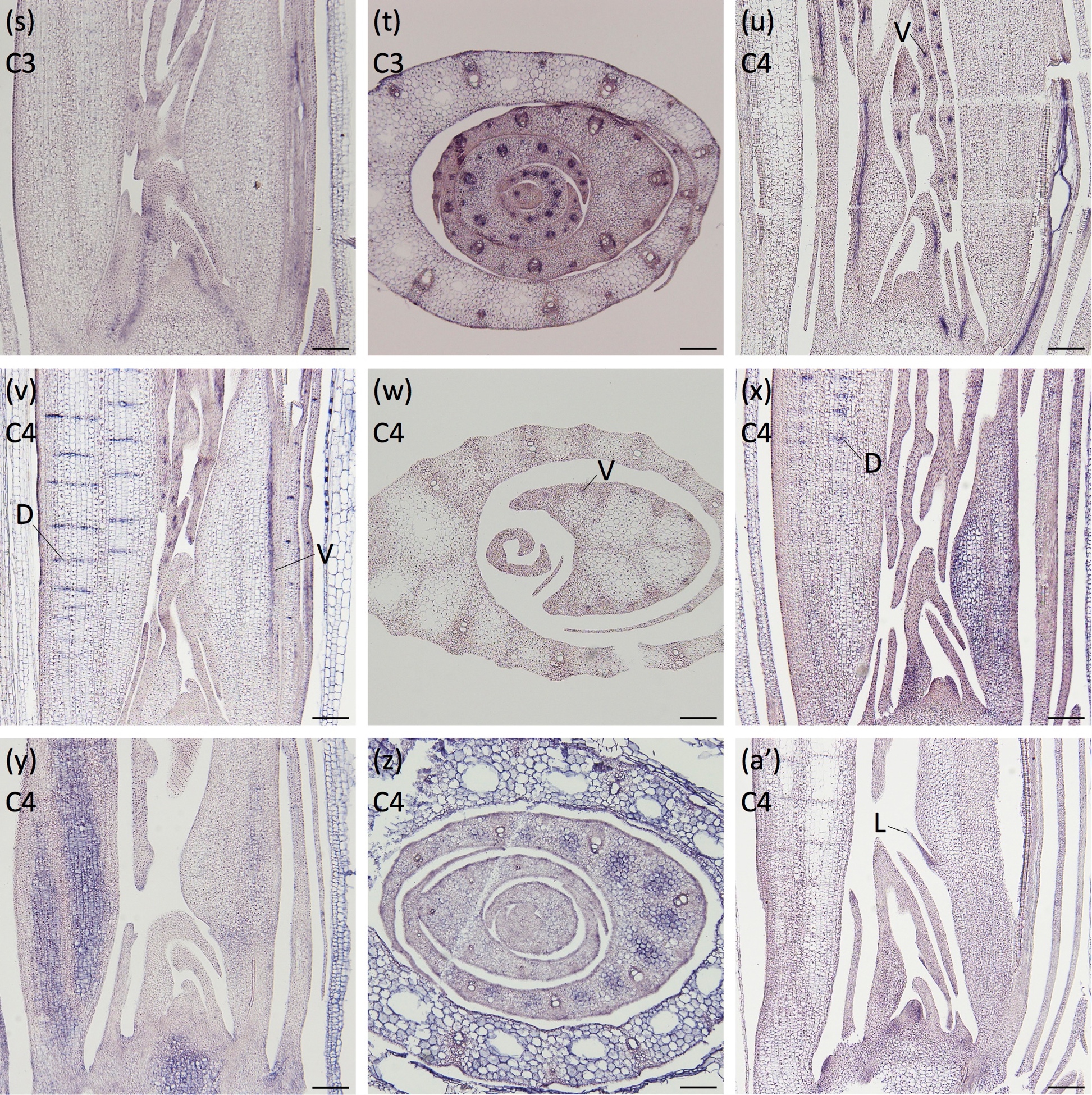


Supplemental Fig. 8. (Continued)

(s, t) *Os04g0690100*, (u) *Os05g0112200*, (v, w) *Os03g0762100*, (x) *Os08g0483900*, (y, z) *Os02g0669900*, and (a’) *Os03g0728900* around the shoot apex.

(s), (u), (v), (x), (y), and (a’) are longitudinal sections of the shoot apices, while (t), (w), and (z) are transverse sections of the shoot apices.

The cluster number to which each gene belongs is indicated in the upper left.

See Supplemental Table 2 for annotation of the genes.

Scale bars: 100 μm.


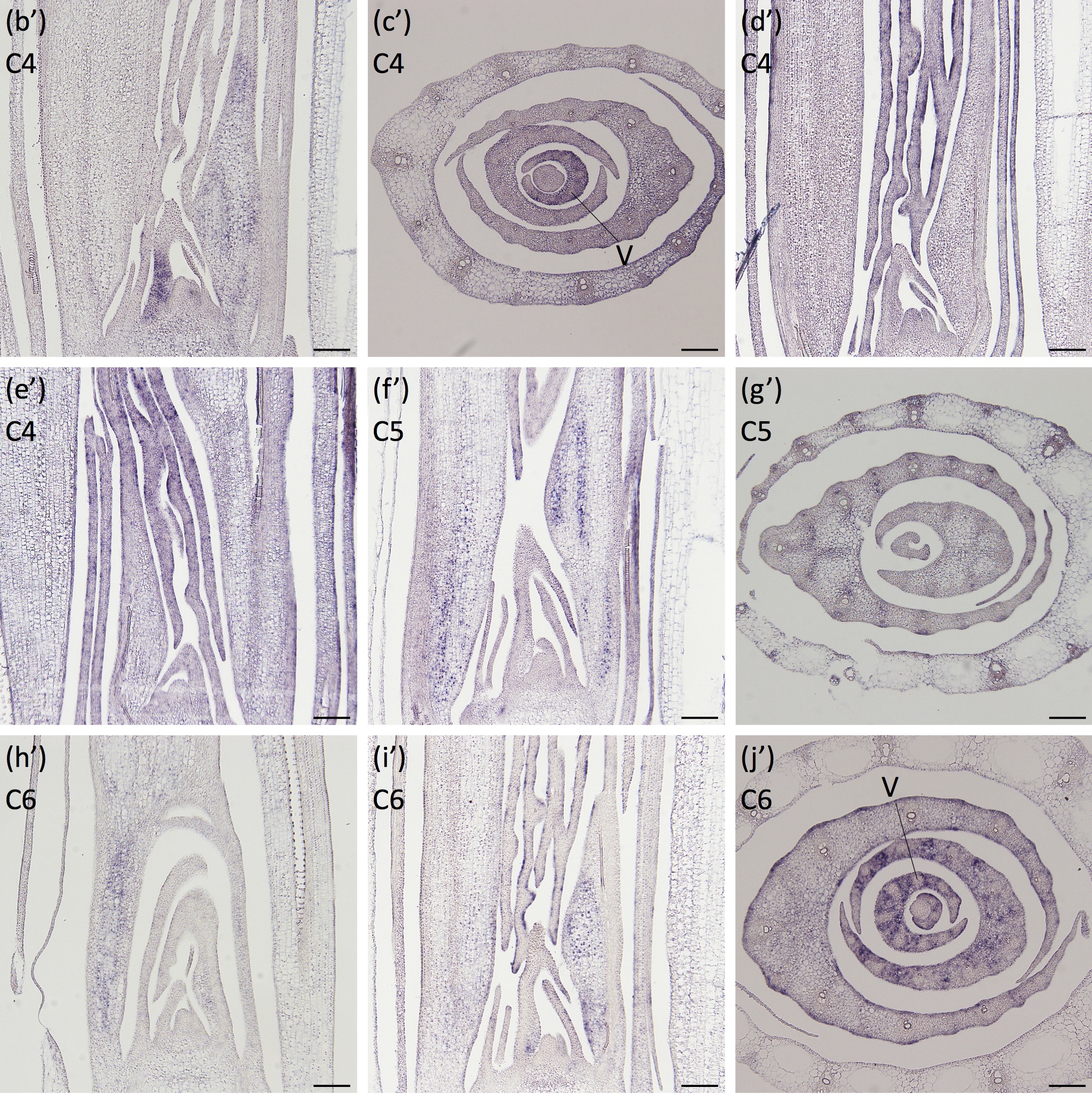


Supplemental Fig. 8. (Continued)

(b’, c’) *Os05g0103000*, (d’) *Os09g0542500*, (e’) *Os03g0127600*, (f’, g’) *Os09g0474100*, (h’) *Os09g0282300*, and (i’, j’) *Os09g0510500* around the shoot apex.

(b’), (d’), (e’), (f’), (h’), and (i’) are longitudinal sections of the shoot apices, while (c’), (g’), and (j’) are transverse sections of the shoot apices.

The cluster number to which each gene belongs is indicated in the upper left.

See Supplemental Table 2 for annotation of the genes.

Scale bars: 100 μm.


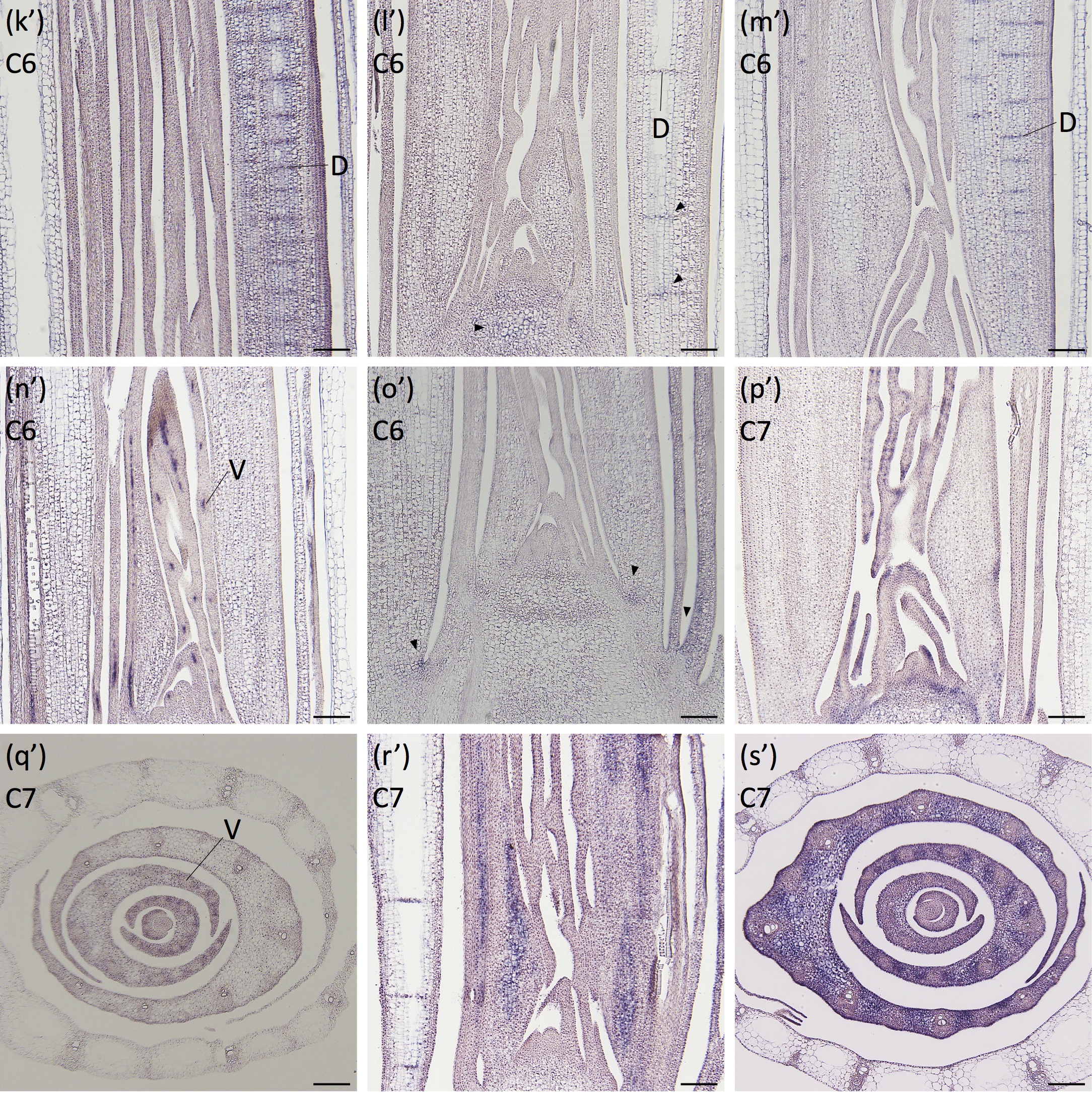


Supplemental Fig. 8. (Continued)

(k’) *Os10g0404900*, (l’) *Os03g0188400*, (m’) *Os08g0260400*, (n’) *Os03g0591300*, (o’) *Os02g0791300*, (p’, q’) *Os06g0724800*, and (r’, s’) *Os03g0135700* around the shoot apex.

(k’) to (p’), and (r’) are longitudinal sections of the shoot apices, while (q’) and (s’) are transverse sections of the shoot apices.

The cluster number to which each gene belongs is indicated in the upper left.

See Supplemental Table 2 for annotation of the genes.

Scale bars: 100 μm.


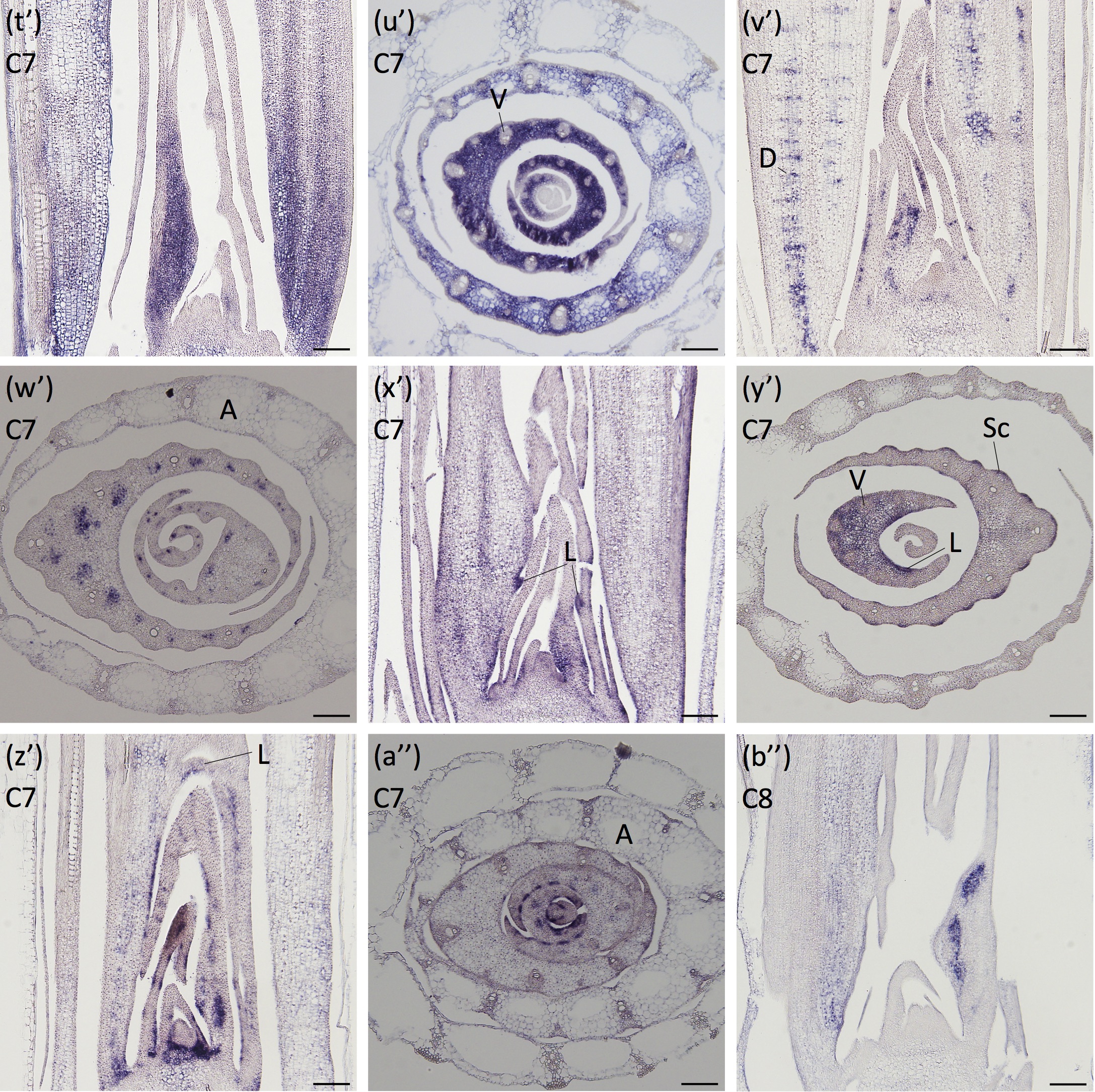


Supplemental Fig. 8. (Continued)

(t’, u’) *Os09g0484200*, (v’, w’) *Os07g0676600*, (x’, y’) *Os01g0848400*, (z’, a’’) *Os08g0444500*, and (b’’) *Os10g0544200* around the shoot apex.

(t’), (v’), (x’), (z’), and (b’’) are longitudinal sections of the shoot apices, while (u’), (w’), (y’), and (a’’) are transverse sections of the shoot apices.

The cluster number to which each gene belongs is indicated in the upper left.

See Supplemental Table 2 for annotation of the genes.

Scale bars: 100 μm.

Supplemental Table 2. Annotation of genes in Fig. 4, 5, Supplemental Fig. 8.

| Figure | RAP ID | RAP Description | Gene Symbol |
| --- | --- | --- | --- |
| Fig. 4 (a, b) | *Os02g0172800* | Similar to Zinc-binding protein. |  |
| Fig. 4 (c) | *Os08g0536800* | Similar to TA1 protein (Fragment). | *OsbHLH080* |
| Fig. 4 (d, e) | *Os03g0338400* | Basic helix-loop-helix dimerisation region bHLH domain containing protein. | *OsbHLH166* |
| Fig. 4 (f) | *Os05g0363500* | Hypothetical conserved gene. |  |
| Fig. 4 (g, h) | *Os05g0500900* | Similar to Indole-3-acetic acid-amido synthetase GH3.5 (EC 6.3.2.-) (Auxin- responsive GH3-like protein 5) (AtGH3-5). | *OsGH3-4* |
| Fig. 5 (a) | *Os02g0164900* | Similar to Auxin response factor 3. | *OsARF6* |
| Fig. 5 (b) | *Os04g0671900* | Transcription factor, Regulator for phosphate homeostasis | *OsARF12* |
| Fig. 5 (c) | *Os06g0677800* | ARF transcription factor, Defense against different types of plant viruses | *OsARF17* |
| Fig. 5 (d) | *Os12g0613700* | Transcriptional factor B3 family protein. | *OsARF25* |
| Fig. S8 (a) | *Os05g0223200* | Similar to Glycine-rich RNA-binding protein GRP2A. |  |
| Fig. S8 (b) | *Os05g0153200* | Uncharacterised domain XH domain containing protein. | *OsFDML4* |
| Fig. S8 (c) | *Os10g0579400* | Conserved hypothetical protein. | *OsWRKY2* |
| Fig. S8 (d) | *Os01g0176500* | Conserved hypothetical protein. |  |
| Fig. S8 (e) | *Os01g0765500* | Conserved hypothetical protein. |  |
| Fig. S8 (f) | *Os02g0258200* | Similar to high mobility group family. |  |
| Fig. S8 (g) | *Os03g0184500* | Transcriptional factor B3 family protein. |  |
| Fig. S8 (h) | *Os04g0228100* | Similar to H0209A05.2 protein. |  |
| Fig. S8 (i) | *Os09g0556800* | Conserved hypothetical protein. |  |
| Fig. S8 (j, k) | *Os11g0149100* | AT-hook motif and PPC domain containing protein, Drought tolerance, Regulation of root development under drought condition, Oxidative stress response, Regulation of the content of chlorophyll | *OsAHL1* |
| Fig. S8 (l) | *Os04g0664400* | Auxin response factor, Transcriptional factor, Transcriptional regulation of plant height and leaf angle | *OsMP* |
| Fig. S8 (m) | *Os03g0244600* | Similar to AUX1-like protein. | *OsLAX2* |
| Fig. S8 (n) | *Os03g0680700* | Similar to Knotted1-interacting protein. |  |
| Fig. S8 (o) | *Os03g0164300* | Conserved hypothetical protein. |  |
| Fig. S8 (p,q) | *Os06g0474500* | Protein of unknown function DUF239, plant domain containing protein. |  |
| Fig. S8 (r) | *Os02g0528100* | Protein of unknown function DUF662 family protein. |  |
| Fig. S8 (s, t) | *Os04g0690100* | Zinc finger, C2H2-type domain containing protein. |  |
| Fig. S8 (u) | *Os05g0112200* | Similar to Zn finger protein (Fragment). | *OsDof19* |
| Fig. S8 (v, w) | *Os03g0762100* | Conserved hypothetical protein. |  |
| Fig. S8 (x) | *Os08g0483900* | Helix-loop-helix DNA-binding domain containing protein. | *OsbHLH047* |
| Fig. S8 (y, z) | *Os02g0669900* | Similar to H0211B05.8 protein. |  |
| Fig. S8 (a’) | *Os03g0728900* | Helix-loop-helix DNA-binding domain containing protein. | *OsbHLH084* |
| Fig. S8 (b’, c’) | *Os05g0103000* | Similar to MYC1. | *OsbHLH083* |
| Fig. S8 (d’) | *Os09g0542500* | Domain of unknown function DUF632 domain containing protein. |  |
| Fig. S8 (e’) | *Os03g0127600* | Forkhead-associated domain containing protein. | *OsDLN74* |
| Fig. S8 (f’, g’) | *Os09g0474100* | Similar to TA1 protein (Fragment). | *OsbHLH085* |
| Fig. S8 (h’) | *Os09g0282300* | Zinc finger, C2H2-type/integrase, DNA-binding domain containing protein. |  |
| Fig. S8 (i’, j’) | *Os09g0510500* | Basic helix-loop-helix (bHLH) transcriptional activator, Control of leaf angle and grain size | *OsBC1* |
| Fig. S8 (k’) | *Os10g0404900* | Similar to HAHB-5 (Fragment). | *OsHox23* |
| Fig. S8 (l’) | *Os03g0188400* | Helix-loop-helix DNA-binding domain containing protein. | *OsbHLH044* |
| Fig. S8 (m’) | *Os08g0260400* | Conserved hypothetical protein. | *OsbHLH171* |
| Fig. S8 (n’) | *Os03g0591300* | Helix-loop-helix DNA-binding domain containing protein. | *OsbHLH140* |
| Fig. S8 (o’) | *Os02g0791300* | Hypothetical conserved gene. | *OsbHLH168* |
| Fig. S8 (p’, q’) | *Os06g0724800* | Basic helix-loop-helix dimerisation region bHLH domain containing protein. | *OsbHLH155* |
| Fig. S8 (r’, s’) | *Os03g0135700* | Basic helix-loop-helix transcription factor, Nucleotide-binding site leucine-rich repeat (NLR) protein-mediated resistance to rice blast disease | *OsRAI1* |
| Fig. S8 (t’, u’) | *Os09g0484200* | Hypothetical protein. |  |
| Fig. S8 (v’, w’) | *Os07g0676600* | HLH (helix-loop-helix) protein, Mediation of defense to brown planthopper (BPH) | *OsbHLH163* |
| Fig. S8 (x’, y’) | *Os01g0848400* | BEL1-type homeobox family, Paralog of RI, Seed shattering, Initiation and maintenance of the shoot apical meristem during embryogenesis, Construction of inflorescence architecture | *qSH1* |
| Fig. S8 (z’, a’’) | *Os08g0444500* | Conserved hypothetical protein. |  |
| Fig. S8 (b’’) | *Os10g0544200* | Basic helix-loop-helix dimerisation region bHLH domain containing protein. | *OsbHLH004* |


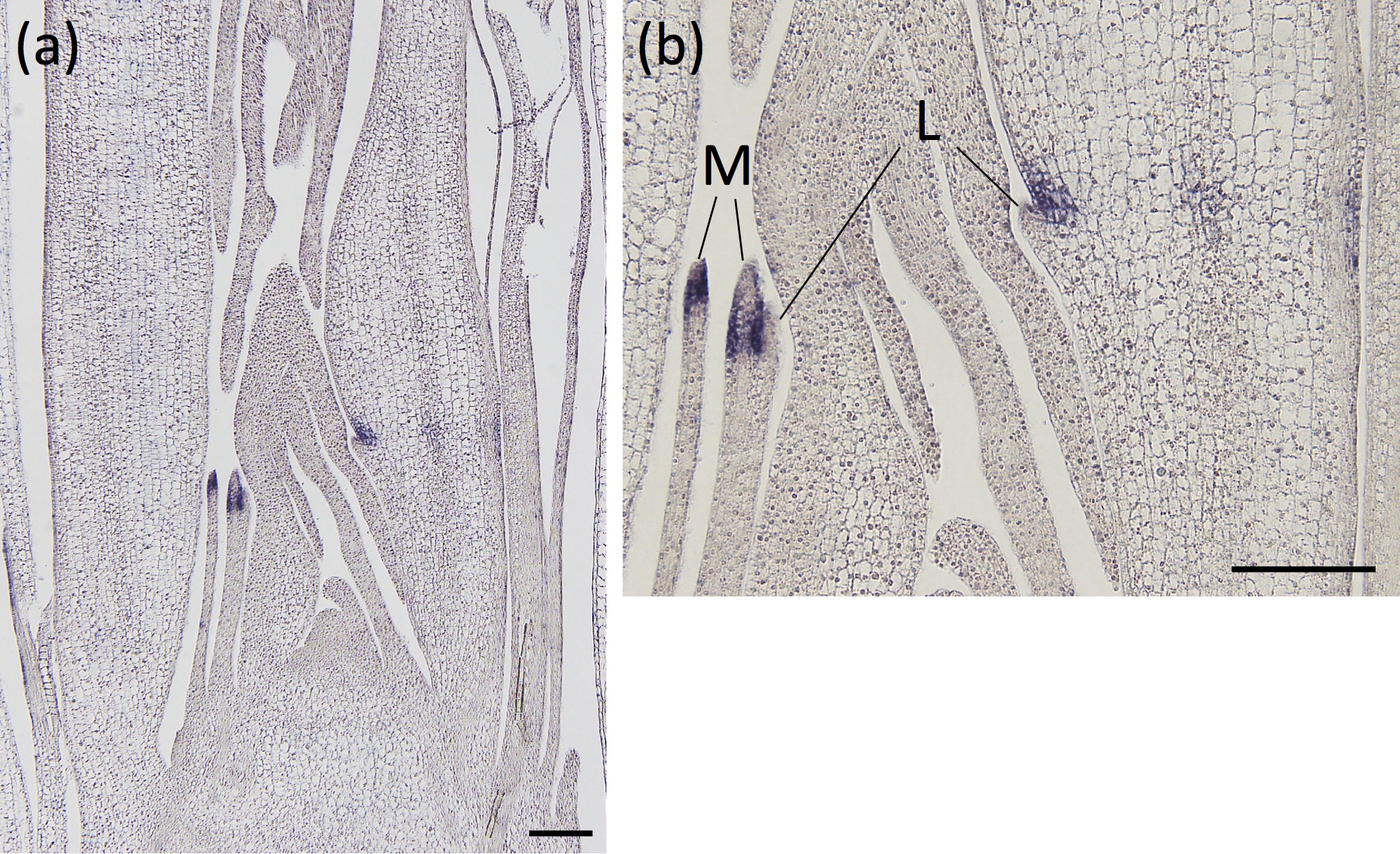


Supplemental Fig. 9. Spatial expression pattern of *LIGULELESS1*.

(a) Expression around the shoot apex in a longitudinal section. (b) Magnified view of (a) at the blade-sheath boundary.

L, ligule; M, marginal part of the blade-sheath boundary. Scale bars: 100 μm.
