# Supplementary material for: Genome-wide analysis of spatiotemporal expression patterns during rice leaf development
Source: BMC Genomics. 2021 Mar 9;22:169. doi: 10.1186/s12864-021-07494-5 (PMC7941727; doi:10.1186/s12864-021-07494-5)
Supplement: Supplementary file 3 — Additional file 3:. Three-dimensional model of Supplemental Figure 4. A three-dimensional model of PCA score plot of samples based on the original principal components. The proportions of the total variance explained by PC1, PC2, and PC3 are shown in parentheses. Samples collected at the same stage are shown in the same color. Samples with different tissue identities are indicated by different symbols: shoot apex, square; P3 leaf, circle; blade, triangle; blade-sheath boundary, diamond; sheath, inverted triangle. Red arrows represent the directions of the modified principal components (mPC1, mPC2, and mPC3) shown in Fig. 2. [file 12864_2021_7494_MOESM3_ESM.html]

 
RGL model


You must enable Javascript to view this page properly.

  
Drag mouse to rotate model. Use mouse wheel or middle button
to zoom it.

---

  
Object written from rgl 0.100.54 by writeWebGL.
